# Variability of strain engraftment and predictability of microbiome composition after fecal microbiota transplantation across different diseases

**DOI:** 10.1038/s41591-022-01964-3

**Published:** 2022-09-15

**Authors:** Gianluca Ianiro, Michal Punčochář, Nicolai Karcher, Serena Porcari, Federica Armanini, Francesco Asnicar, Francesco Beghini, Aitor Blanco-Míguez, Fabio Cumbo, Paolo Manghi, Federica Pinto, Luca Masucci, Gianluca Quaranta, Silvia De Giorgi, Giusi Desirè Sciumè, Stefano Bibbò, Federica Del Chierico, Lorenza Putignani, Maurizio Sanguinetti, Antonio Gasbarrini, Mireia Valles-Colomer, Giovanni Cammarota, Nicola Segata

**Affiliations:** 1 Digestive Disease Center, Fondazione Policlinico Universitario ‘A. Gemelli’ IRCCS, Rome, Italy; 2 Department of Translational Medicine and Surgery, Catholic University of Rome, Rome, Italy; 3 Department CIBIO, University of Trento, Trento, Italy; 4 Microbiology Unit, Fondazione Policlinico Universitario ‘A. Gemelli’ IRCCS, Rome, Italy; 5 Department of Basic Biotechnological Sciences, Intensivological and Perioperative Clinics, Catholic University of Rome, Rome, Italy; 6 Department of Diagnostic and Laboratory Medicine, Unit of Parasitology and Multimodal Laboratory Medicine Research Area, Unit of Human Microbiome, Bambino Gesù Children’s Hospital IRCCS, Rome, Italy; 7 IEO, Istituto Europeo di Oncologia IRCSS, Milan, Italy

**Keywords:** Metagenomics, Applied microbiology

## Abstract

Fecal microbiota transplantation (FMT) is highly effective against recurrent *Clostridioides difficile* infection and is considered a promising treatment for other microbiome-related disorders, but a comprehensive understanding of microbial engraftment dynamics is lacking, which prevents informed applications of this therapeutic approach. Here, we performed an integrated shotgun metagenomic systematic meta-analysis of new and publicly available stool microbiomes collected from 226 triads of donors, pre-FMT recipients and post-FMT recipients across eight different disease types. By leveraging improved metagenomic strain-profiling to infer strain sharing, we found that recipients with higher donor strain engraftment were more likely to experience clinical success after FMT (*P* = 0.017) when evaluated across studies. Considering all cohorts, increased engraftment was noted in individuals receiving FMT from multiple routes (for example, both via capsules and colonoscopy during the same treatment) as well as in antibiotic-treated recipients with infectious diseases compared with antibiotic-naïve patients with noncommunicable diseases. Bacteroidetes and Actinobacteria species (including *Bifidobacteria*) displayed higher engraftment than Firmicutes except for six under-characterized Firmicutes species. Cross-dataset machine learning predicted the presence or absence of species in the post-FMT recipient at 0.77 average AUROC in leave-one-dataset-out evaluation, and highlighted the relevance of microbial abundance, prevalence and taxonomy to infer post-FMT species presence. By exploring the dynamics of microbiome engraftment after FMT and their association with clinical variables, our study uncovered species-specific engraftment patterns and presented machine learning models able to predict donors that might optimize post-FMT specific microbiome characteristics for disease-targeted FMT protocols.

## Abstract (web summary)

Coupling microbial metagenomics with machine learning enables prediction of donor strain engraftment after fecal microbiota transplantation (FMT) for a range of diseases, and may help tailor design of FMT to optimize microbial engraftment and achieve clinical outcomes.

## Main

Fecal microbiota transplantation (FMT) is the medical procedure of transferring human fecal matter from a healthy donor to a recipient to treat a disease related to microbiome imbalance. FMT has shown nearly 90% success rate for the treatment of recurrent *Clostridioides difficile* infection (rCDI)^1,2^, for which it is approved in clinical practice^3^. FMT has been explored more recently for other diseases associated with microbiome alterations^4–6^ or to support other therapies^7–9^, but its efficacy is usually lower and less consistent over cohorts than for rCDI^10,11^. Some factors that may explain this variability include the presence or abundance of single bacteria and the diversity of the patient microbiome at baseline^5,6^, clinical characteristics of the disease^12^, the composition of the donor’s gut microbiome^13^, specific aspects of the FMT working protocols (for example route of delivery, amount of infused feces)^14^ and differential engraftment among species^5,6^. Yet, it is generally unknown how strain engraftment might be linked with clinical remission after FMT.﻿

The mechanisms and dynamics dictating which donor microbes can engraft in the recipient are poorly understood. Initial studies able to track the transmission of donor strains to the recipient have been performed on very few donor–recipient pairs^15^. Availability of larger FMT trials and the advances in strain-resolved metagenomics enabled deeper analyses that started unraveling the engraftment efficiency of FMT across diseases and led to the development of statistical models to predict the post-FMT microbiome composition^16^. Such investigations remained confined to single cohorts^16–21^, with unanswered questions about cross-cohort and cross-condition generalizability. As deeper strain-level metagenomics is possible^22–24^ and not limited to well characterized microbial taxa^25^, and as more metagenomic datasets are becoming available^7–9,15–18,26–35^, an integrative metagenomic analysis may allow uncovering general patterns of microbial engraftment and connected clinical outcomes.

Here, we present a systematic meta-analysis of 24 studies that investigated FMT in different clinical settings for which we employed new strain-resolved metagenomic approaches to unravel the dynamics of FMT engraftment and its links with clinical outcomes.

## Results

### A meta-analysis of public and new FMT metagenomic datasets

We retrieved all FMT studies with publicly available data that assessed microbiome composition of donors and recipients (pre- and post-FMT) through shotgun metagenomics (Methods). This search yielded a total of 21 datasets (Table 1 and Supplementary Table 1)^7–9,15–18,26–35^. In each study, we removed samples that were not sequenced at sufficient depth (<1 Gbp) or with evidence of mislabeling (Methods). The retained metagenomes belong to 203 FMT procedures for which at least one sample is available from each member of the ‘FMT triad’: the pre-FMT recipient, the post-FMT recipient and the corresponding donor. When multiple post-FMT samples were available, we selected the post-FMT sample collected closest to 1 month after FMT, as 30 days was the value that minimizes the overall time deviation (Supplementary Fig. 1; Methods).

**Table 1:** Summary and main characteristics of the FMT datasets included in this meta-analysis

Disease	No. of datasets (new datasets)	No. of recipients (new recipients)	No. of samples (new samples)	Median no. of post-FMT samples [IQR]	Disease category	Countries
*Clostridioides difficile* infection	9 (1)	96 (16)	529 (94)	2.0 [3.0]	Infectious	Italy, Germany, Norway, USA, Canada
Inflammatory bowel disease	5 (1)	38 (2)	188 (8)	2.0 [1.0]	Chronic	France, Italy, USA
Multidrug-resistant bacteria colonization	3 (1)	21 (5)	109 (13)	1.0 [2.0]	Infectious	Italy, Israel, France, the Netherlands, Switzerland
Melanoma	2	24	248	4 [7]	Oncological	Israel, USA
Tourette syndrome	1	5	25	2.0 [0.0]	Chronic	China
Metabolic syndrome	2	16	154	3 [0.2]	Chronic	the Netherlands
Irritable bowel syndrome	1	20	91	2.0 [0.0]	Chronic	Norway
Tyrosine kinase inhibitor-dependent diarrhea	1	6	27	2.0 [1.5]	Oncological	Italy
**Total**	**24 (3)**	**226 (23)**	**1,371 (116)**	**2 [2]**		

Numbers in parenthesis refer to data collected specifically for the present study.

We additionally sequenced 116 stool samples (23 FMT triads) from three cohorts of patients with rCDI, inflammatory bowel disease (IBD) and clinically relevant colonization by multidrug-resistant bacteria (MDRB) (Table 1 and Supplementary Table 2; Methods) enrolled in prospective case series from Italy (Fondazione Policlinico Gemelli IRCCS and Bambino Gesù Children’s Hospital; Methods) and sequenced at a higher read depth than most existing FMT datasets (Supplementary Figs. 2 and 3).

In total, 1,371 samples and 226 FMT triads from 24 different cohorts (Table 1) were included in the analysis covering nine clinical conditions, including rCDI (*n* = 9), IBD (*n* = 5), MDRB (*n* = 3), melanoma (*n* = 2) and metabolic syndrome (*n* = 2), and single cohorts of irritable bowel syndrome (IBS), Tourette syndrome and diarrhea induced by tyrosine kinase inhibitors^7–9,15–18,26–35^. Studies enrolled adult participants with the exception of HouriganS_2019 (ref. ^29^), ZhaoH_2020 (ref. ^35^), This_study_MDRB and This_study_IBD and originated from countries with Mediterranean (France, Italy, Israel) and Northern European lifestyles (Germany, the Netherlands, Norway), in North America (USA) and China. All samples were processed following the same computational pipeline, from quality-control to analysis by strain-level profiling including yet-to-be-characterized species based on the species-level genome bins (SGBs; Methods) framework^25^. While we used all 1,371 samples (together with 4,443 samples from unrelated longitudinal datasets) to optimally delineate strain identity, we limited the analyses to one post-FMT sample per FMT triad (559 samples).

### Strain-level metagenomics can assess microbial engraftment

To identify the transfer and engraftment of the donor microbiome in the recipient, we exploited the observation that microbial strains are generally specific to individuals and rarely found shared between unrelated individuals^22,23,36^. We adopted an operational species-specific definition of ‘strain’^37,38^ by comparing phylogenetic distance distributions of microbial genetic profiles of a given species sampled from the same individual over multiple timepoints with those distributions obtained comparing profiles from unrelated individuals (Supplementary Table 3; Methods). We implemented the approach and the species-specific cut-offs that define strain identity within StrainPhlAn 4 (ref. ^39^), which we empowered with a custom database of marker gene sequences from around 729,000 microbial genomes and metagenome-assembled genomes (MAGs). With such references, StrainPhlAn is able to detect and model strains belonging to a total of 4,992 yet-to-be-characterized species^25^; that is, unknown SGBs (uSGBs; Methods).

**Fig. 1:**
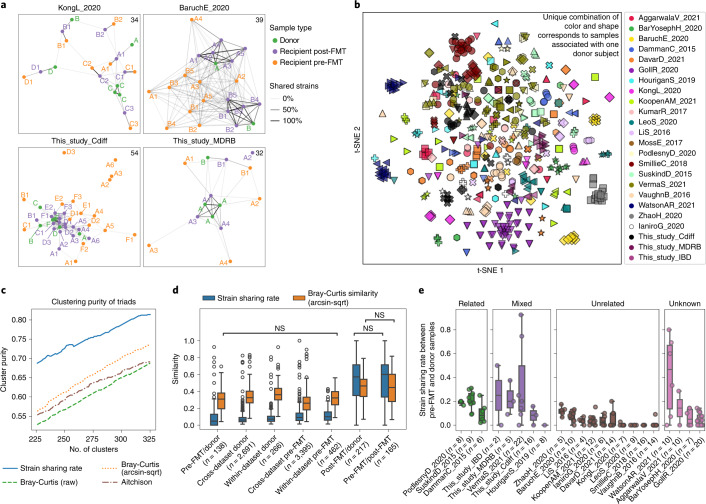
Overview of microbial strain sharing in FMT studies. **a**, Strain-sharing networks of the two new FMT cohorts with *C. difficile* and MDRB colonization and of two published FMT cohorts^9,30^. Nodes represent samples and are colored by role in FMT triads. The letters correspond to the donor subject and letter/number combinations indicate both associated donors (the letter) and FMT instance membership (the number) of pre-/post-FMT samples. Edges report strain sharing (minimum 2) and their opacity is scaled to the maximum number of shared strains in each dataset (indicated in the top right corner). Extended Data Fig. 1 reports the networks of all 24 datasets. **b**, Ordination of samples from all cohorts based on strain sharing rates (t-SNE with perplexity = 20). See Extended Data Fig. 3 for a PCoA ordination. **c**, Strain-sharing enabled more precise reconstructions of the true FMT triads compared with species-level *β*-diversities (Extended Data Fig. 2 and Supplementary Table 5). We compare the K-medoids clustering purity of FMT triads between strain-sharing distances and on Bray–Curtis dissimilarities/Aitchison distances as a function of the number of clusters K. **d**, Strain-sharing rate and Bray–Curtis similarity between pairs of samples show that strain-sharing rates increase much more after FMT compared with Bray–Curtis similarity. Significance was assessed by Mann–Whitney *U*-tests and the two-tailed *P* values were FDR-adjusted using the BH method. All pairwise tests are significant except for those labeled NS. All *P* and *Q* values are reported in Supplementary Table 6. **e**, Distribution of strain-sharing rates between donor and corresponding recipient pre-FMT samples showing that donors share more strains with recipients pre-FMT when the individuals are ‘related’ (same family/household or friends; Methods). Boxplots report the median and upper/lower quartiles, whiskers are at 1.5 times higher/lower of the upper/lower quartiles.

**Extended Data Fig. 1:**
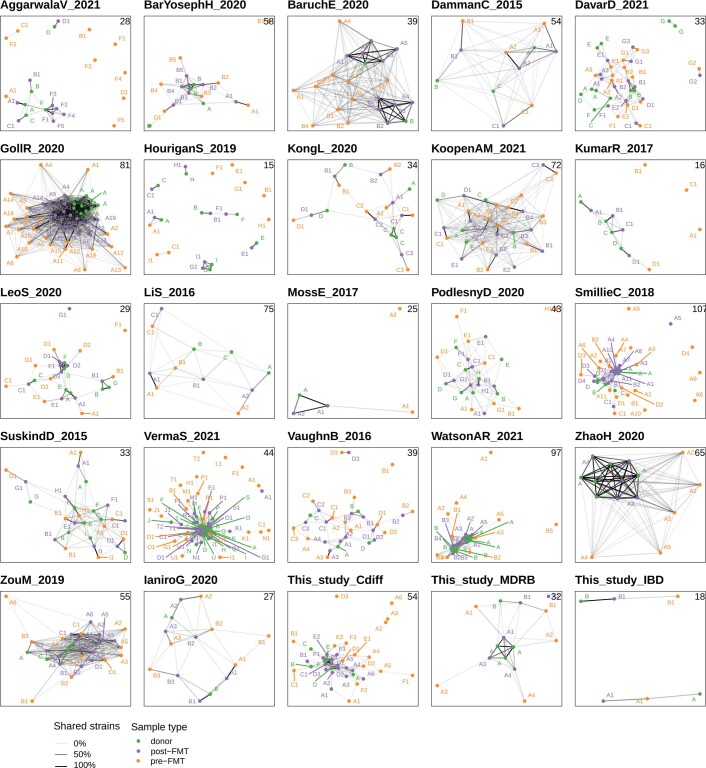
Strain sharing networks for the datasets included in this study not shown in Fig. 1A. Each node corresponds to a sample and is colored by its role in FMT triads (recipient pre-FMT sample, recipient post-FMT sample, and donor's sample). Edge opacity is proportional to the number of shared strains between two samples (Methods*)* and only edges corresponding to at least 2 shared strains are shown. The structure of the networks illustrates how FMT triads tend to cluster together but with different clustering characteristics across cohorts.

The StrainPhlAn-based pipeline allowed generating a map of between-samples strain sharing events that we recapitulated in undirected networks based on the number of common strains between samples (Fig. 1a and Extended Data Fig. 1; Methods). These networks confirmed that samples from the same FMT triad tend to share many more strains than unrelated individuals, whereas they are connected only weakly to samples of other FMT triads in the same cohort (Fig. 1a, PERMANOVA by FMT triad and dataset on strain sharing-based dissimilarity metric, *R*^2^ = [0.05–0.61] and *Q* < 0.1 in 14 of the 24 datasets; Supplementary Table 4).

**Extended Data Fig. 2:**
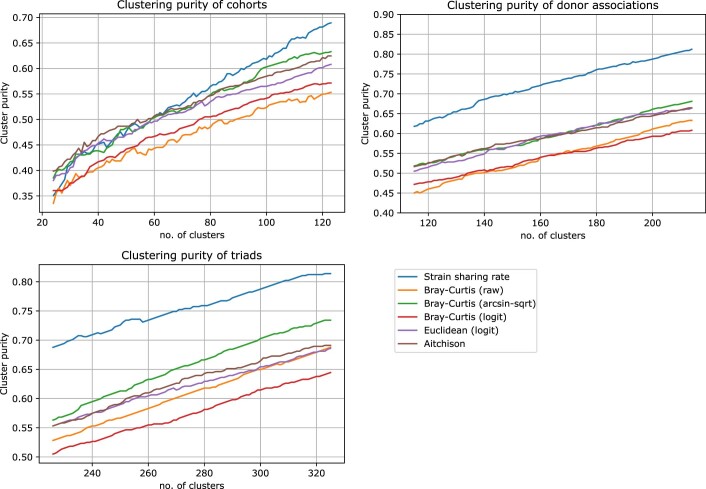
The purity of K-medoids clustering with varying K shows that strain sharing rate outperforms *β*-diversity measures in clustering by donor associations and by FMT triads. In clustering by cohorts for the low number of clusters it gets outperformed by Aitchison distance, but catches up as the K increases.

**Extended Data Fig. 3:**
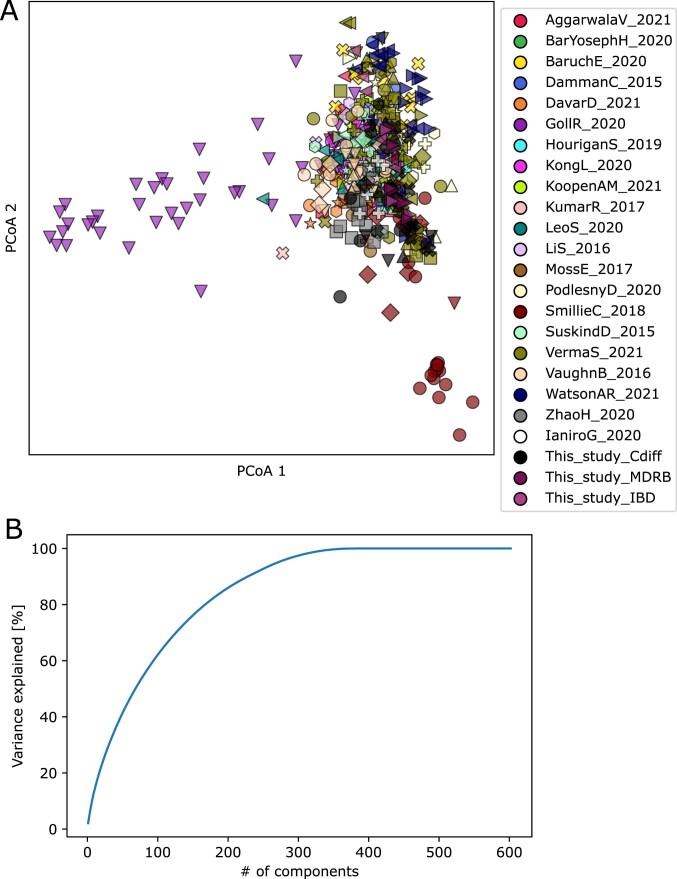
PCoA ordination on strain sharing rate distances and variance explained by number of components, suggesting that two dimensions are not sufficient to linearly separate the clusters induced by dataset or donor batch effects. Unique combinations of color and shape correspond to samples associated with one donor subject.

To account for different numbers of strains that can be analyzed over samples, we defined the strain-sharing rate metric as the number of strains found identical in two samples divided by the number of species with available strain profiles that are present in both samples (Methods). K-medoids clustering on strain-sharing rates yielded clusters of higher purity with respect to FMT triad membership than *β*-diversity measures (Fig. 1c, Extended Data Fig. 2 and Supplementary Table 5; Methods) and a t-distributed stochastic neighbor embedding (t-SNE) projection also separates samples by FMT triad membership (Fig. 1b and Extended Data Fig. 3). Strain-level metagenomics can thus accurately describe strain sharing events within FMT triads.

### Donor–recipient relationship influences post-FMT engraftment

Strain-sharing rates were much higher between post-FMT and donor samples (median 57%), and between pre-FMT and post-FMT samples (60%) than between donors and pre-FMT recipients (4.8%). The substantial increase in donor–recipient strain sharing after FMT is also significantly stronger than the decrease in *β*-diversity (Wilcoxon signed-rank test, *P* = 7 × 10^−23^; Fig. 1d and Supplementary Table 6; Methods), confirming that the strain identity-based profiling approach better captures the microbiome remodeling induced by FMT compared with species-level *β*-diversity measures.

**Extended Data Fig. 4:**
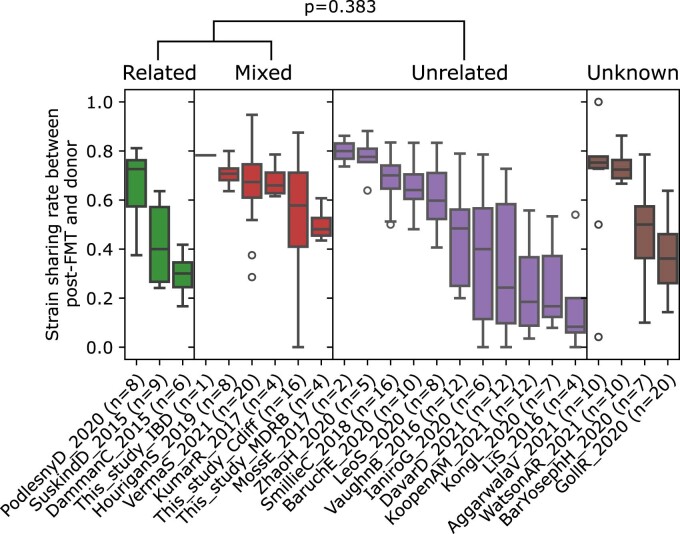
Strain sharing rates between donor and post-FMT samples is non-significantly higher in datasets using related or a mixture of related and unrelated donors compared to those using only unrelated donors (related or mixed vs unrelated, permutation test, p=0.383). Box plots are defined as follows: the center line and upper and lower limit of the box correspond to the median, upper quartile and lower quartile respectively. The whiskers are defined by that data point that is at most 1.5 times higher than the upper quartile (upper whisker) or 1.5 times lower than the lower quartile (lower whisker).

Overall, 58.4% of post-FMT samples shared more strains with corresponding donor samples than with their pre-FMT. However, the difference in shared strains between donor/post-FMT samples and pre-FMT/post-FMT samples differed substantially across FMT triads (median = −3; range = −96–75; Extended Data Fig. 1 and Supplementary Fig. 4). We also found that pre-FMT recipients shared more strains with related (usually cohabitating) donors than with unrelated donors (that is, donors in the original studies that were specified as unrelated, or recruited through public advertisement or hospital cohorts (Fig. 1e), related versus unrelated, permutation test *P* < 1 × 10^–4^, median strain sharing rate difference = 0.18). This also holds in datasets in which only a subset of the donors and recipients were related (Fig. 1e; *P* < 1 × 10^–4^). We accounted for these potential baseline strain sharing biases by subtracting them from post-FMT engraftment rates, resulting in significantly lower estimates (Wilcoxon signed-rank test, p = 1 × 10^–9^; Supplementary Fig. 5 and Extended Data Fig. 4; Methods). Together, these data confirm that the extent of donor microbiome engraftment is variable and influenced by pre-FMT donor–recipient relatedness.

### Combined FMT administration associates with strain engraftment

**Fig. 2:**
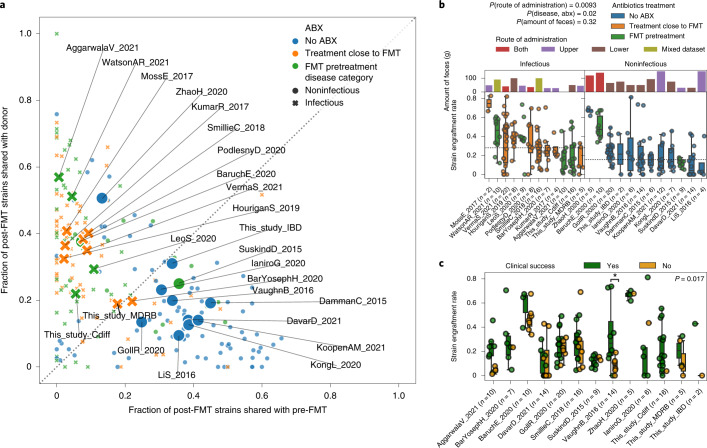
Variability of strain engraftment and retention across disease, antibiotics use and clinical success. **a**, Distribution of the fraction of donor strains and the fraction of retained strains present in the post-FMT samples for all FMT triads, showing a separation between higher fraction of donor strains and higher fraction of retained strains that associates with antibiotics administration and disease category. Small points represent individual FMT triads and large labeled marks represent per dataset averages. **b**, Variability of strain engraftment rate by disease category, antibiotics usage and route and amount of administered feces, highlighting the complex association between these variables and strain engraftment rates. The horizontal line is the median of per dataset medians. The statistical tests are performed by permuting the variables associated with datasets (two-tailed permutation test route of administration mixed versus lower or upper *P* = 0.0093, antibiotics and infectious disease versus no antibiotics and noninfectious disease in datasets employing single route of administration *P* = 0.02, amount of feces *P* = 0.32). **c**, Association between clinical success of FMT and strain engraftment rates for the 13 studies in which the information on clinical success was available and for which at least one recipient was in each group. The definition of clinical success for each study is reported in Supplementary Table 1. Permutation tests with success labels permuted within each dataset pointed at an overall significant association of strain engraftment with clinical success (two-tailed *P* = 0.017), that was significant in only 1 of the 13 datasets when considered individually (VaughnB_2016 Mann–Whitney *U*-test with two-tailed *P* = 0.039). Boxplots plots report the median and upper/lower quartiles, whiskers are at 1.5 times higher/lower of the upper/lower quartiles.

**Extended Data Fig. 5:**
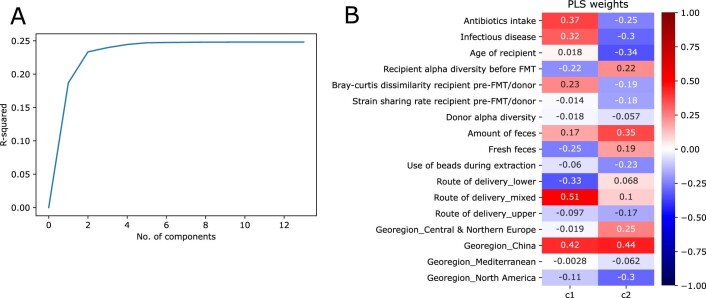
Partial least squares regression of various variables of interest against strain engraftment rate. A) Most of the explained variance in strain engraftment rate is covered by the first two components. B) The weights of the variables in the first two components.

To assess the main determinants of post-FMT strain engraftment, we first performed a multivariate analysis including clinical variables that could potentially influence engraftment (infectious/noninfectious disease, antibiotics treatment), recipient and donor microbiome characteristics (*α*-diversity, species-level dissimilarity and strain sharing rate at baseline, recipient age and geographical region) and other procedural features that were consistently available across datasets (administration of fresh/frozen stool, amount of feces administered, route of administration and bead-beating steps in the DNA extraction protocol; Methods). By fitting a partial least squares (PLS) regression model (Methods), we found that only the first two components were significantly associated with engraftment, explaining 18.7% (*Q* = 6 × 10^–10^) and 4.6% (*Q* = 3.8 × 10^–3^) of the variation (Extended Data Fig. 5). Only FMT administration through a mixed route combining upper gastrointestinal tract administration (by capsules, enteroscopy, nasogastric tube, nasoduodenal tube or upper endoscopy) and lower gastrointestinal tract administration (by colonoscopy) was significantly associated with the first PLS component (*P* = 0.016). Indeed, route of delivery emerged as the variable most significantly associated with strain engraftment also in univariate testing (*P* = 0.0093; Fig. 2b). So far, no consensus exists as to a recommended route of administration in FMT protocols^40^ and, whereas our results suggest that combined routes increase the engraftment likelihood, the observation is based on only four studies adopting this approach. Importantly, intake of antibiotics (14 studies with antibiotic intake before FMT, 10 without) and disease category (12 studies on infectious diseases, 12 on noninfectious) were significantly associated with strain engraftment in cohorts that employed a single administration route (*n* = 19 datasets, permutation test antibiotics treatment and infectious disease versus no antibiotics and noninfectious disease, *P* = 0.027), and both were associated with the first two PLS components while being highly correlated with each other (Supplementary Fig. 6; Methods).

### FMT engraftment is linked to antibiotics and infectious diseases

We examined the extent of donor strain engraftment over strain retention in FMT recipients by comparing the fraction of donor strains detectable in the post-FMT sample (fraction of donor strains) with the fraction of pre-FMT strains detectable in the post-FMT sample (fraction of retained strains; Fig. 2a). We found that patients who received antibiotics before FMT—as part of their therapy for underlying diseases or as pretreatment before FMT—had a significantly higher fraction of donor strains compared with the fraction of retained strains, as was previously reported in the context of ulcerative colitis^41^ (Wilcoxon signed-rank test, *P* = 2 × 10^–16^), while the opposite was true for recipients who did not receive antibiotics (Wilcoxon signed-rank test, *P* = 1 × 10^–5^, Fig. 2a). Antibiotic treatment thus seems to lead to enhanced donor strain engraftment and decreased strain retention in the FMT recipient, possibly by reducing colonization resistance in the recipient^42^. Recipients with infectious diseases also had comparatively higher fractions of donor strains compared with the fraction of retained strains (Wilcoxon signed-rank test, *P* = 8 × 10^–16^), while the opposite was true in patients with noninfectious diseases (Wilcoxon signed-rank test, *P* = 6 × 10^–4^).

Patients with recurrent or resistant infectious diseases often have a long history of repeated antibiotic courses and are pretreated with specific antibiotics before FMT, while only two of the noninfectious disease cohorts (SuskindD_2015 and BaruchE_2020) underwent treatment with antibiotics before FMT. The SuskindD_2015 cohort of patients with Crohn’s disease received rifaximin before FMT and exhibits strain sharing patterns similar to datasets with noninfectious disorders, consistent with previous results showing that rifaximin does not lead to substantial shifts in microbiome composition^43^. On the contrary, the BaruchE_2020 melanoma cohort, in which patients were pretreated with neomycin and vancomycin, displayed strain sharing characteristics similar to cohorts with infectious diseases treated with antibiotics, possibly due to the disruptive effect of combined oral vancomycin^44^ and neomycin^45^ treatment. Antibiotic use may also explain the successful engraftment observed in patients with infectious diseases treated with artificial microbiome consortia^46^.

Administration of stool samples from multiple donors could also maximize the diversity of engrafted bacteria in the recipient^47^. For the only study available adopting mixed donor feces (GollR_2020), we found the second highest median strain engraftment rate among the cohorts without antibiotics treatment. We also observed an exceptionally high microbial strain sharing between donors and post-FMT recipients, comparable with datasets of infectious diseases and pre-FMT antibiotics, in the ZhaoH_2020 cohort, where FMT was given for Tourette syndrome (a noninfectious disorder) without antibiotic preconditioning. Besides using a mixed administration route, this cohort included children whose microbiome is less resistant to colonization from incoming strains.

Overall, these results show that the fractions of donor-derived and retained strains after FMT are influenced by antibiotic administration and by the presence of an infectious disease, which are both hypothesized to reduce microbiome colonization resistance. However, since antibiotic treatment and infectious diseases were closely entangled variables in our meta-cohort (Supplementary Fig. 6), it was not possible to unravel their relative contribution to strain engraftment and retention. Nonetheless, as both variables are known to lead to a decreased microbial diversity^48,49^ and, given that the substantially lower microbial *α*-diversity is probably making the recipient’s gut more receptive to foreign strains from the donor, we hypothesize that these factors may have a combined effect on the overall engraftment.

### Links between strain engraftment and clinical success of FMT

Previous studies suggest that strain engraftment might be associated with clinical success of FMT, but consolidated evidence is still lacking^5,6^. We thus compared the strain engraftment rates with the clinical success of each FMT triad for the datasets with appropriate clinical data available (Supplementary Table 1; Methods). When considering single studies, we found that recipients experiencing clinical success showed significantly higher engraftment only in the VaughnB_2016 cohort (Mann–Whitney *U*-test, *P* = 0.039). When analyzing all cohorts together, we found an overall positive association between strain engraftment rate and clinical response to FMT (Fig. 2c) that proved significant according to a blocked permutation test and a Wilcoxon signed-rank test on medians (*P* = 0.017 and *P* = 0.040, respectively) and borderline significant using a random effects model meta-analysis (*P* = 0.051; Methods). We similarly tested for an association between the species-level similarity between post-FMT and donor samples and clinical success, which yielded a significantly positive association when evaluating species-level microbial abundances with a blocked permutation test (Bray–Curtis similarity between post-FMT and donor samples *P* = 0.018) but not with the other tests (random effects model meta-analysis *P* = 0.072, Wilcoxon signed-rank test on medians *P* = 0.414; Supplementary Fig. 7), and no significant association was found when considering overlap in species presence (Jaccard similarity between post-FMT and donor samples; Supplementary Fig. 8). The limited total sample size, the binary categorization of success of clinical treatments, and the heterogeneity of conditions tested represent limitations in our analyses, but the results overall suggest that both higher microbial engraftment and, partially, the overall convergence of microbial species abundances between recipient and donor might improve clinical success of FMT.

### Post-FMT strain engraftment rates are phylum- and species-dependent

**Fig. 3:**
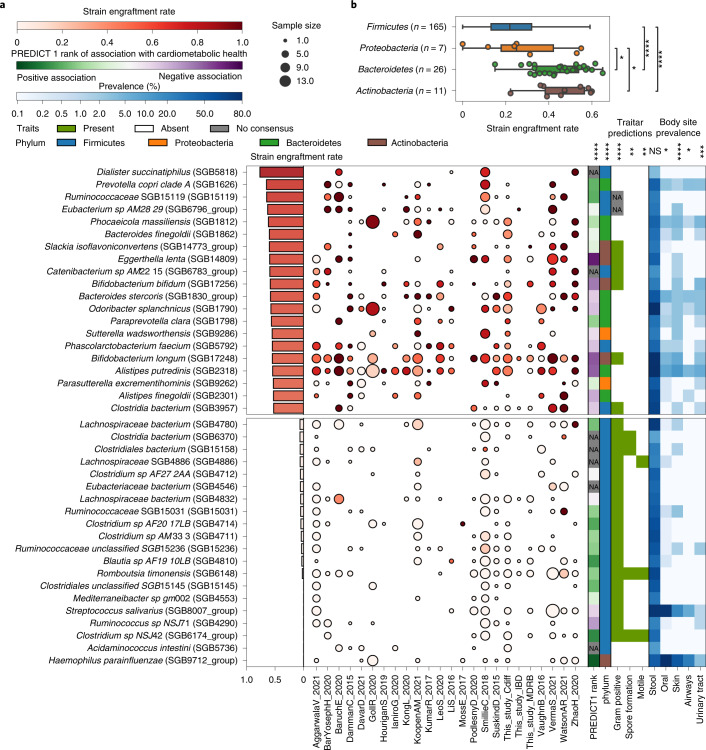
Bacterial strain engraftment rates across datasets and their associations with phenotypic properties, cardiometabolic health and prevalence. **a**, Overall and within-dataset strain engraftment rates and associations of species with predicted phenotypic properties^62^, cardiometabolic health^53^ and prevalence (%) in different human body sites. Overall strain engraftment rate is computed over all triads. Out of 211 species assessed (Supplementary Table 8), the 20 species displaying highest and lowest engraftment rates are reported. Associations with continuous variables were tested with Spearman’s rank correlation tests, while those with binary categorical variables were tested with the Mann–Whitney *U*-test. The association with phylum was tested with the Kruskal–Wallis test. Tests were performed for all species including those not shown, and *P* values were FDR corrected using the BH method (Supplementary Table 10). Significance levels (NS, nonsignificant, *Q < 0.05, **Q < 0.01, ***Q < 0.001, ****Q < 1 × 10^–4^) are reported above each metadata column. Sample size is defined as the number of FMT triads in which the species could engraft as defined by the strain engraftment rate measure (Methods). **b**, Strain engraftment rates are significantly associated with bacterial phyla (Kruskal–Wallis test, *P* = 3 × 10^–11^; post hoc Dunn tests FDR corrected using the BH method, Firmicutes versus Bacteroides *Q* = 8.0 × 10^–9^, Firmicutes versus Actinobacteria *Q* = 3 × 10–5, Proteobacteria versus Bacteroidetes *Q* = 0.037, Proteobacteria versus Actinobacteria *Q* = 0.037, the remaining pairs are NS, that is *Q* > 0.1). The Euryarchaeota and Verrucomicrobia phyla were omitted from the analysis as only one species in each of those phyla was assessed in our analysis. Boxplots report the median and upper/lower quartiles, whiskers are at 1.5 times higher/lower of the upper/lower quartiles. NA, not applicable.

We then computed species-specific strain engraftment rates over all FMT triads for the 211 microbial species for which the strain engraftment rate could be estimated with sufficient confidence (that is, that could engraft in at least 15 FMT triads and four different datasets; Fig. 3a and Supplementary Table 7; Methods). Overall, we found significant differences in engraftment rates across bacterial phyla (Kruskal–Wallis test, *P* = 3 × 10^–11^), as Bacteroidetes and Actinobacteria spp. (26 and 11 species, respectively) displayed higher average strain engraftment rates (45 ± 12% and 46 ± 12%, respectively; Fig. 3b and Supplementary Table 8) compared with Firmicutes and Proteobacteria (23 ± 14% and 29 ± 20%, respectively; post hoc Dunn tests, *Q* < 0.1; Fig. 3b).

Six Firmicutes SGBs were among the set of the 20 most-engrafting species, including two species with only a few isolate genomes available (*Dialister succinatiphilus*, *Phascolarctobacterium faecium*), two SGBs belonging to hitherto undescribed species (*Eubacterium* SGB6796, *Catenibacterium* SGB6783), and two others belonging to genera without cultured representatives (*Clostridia* SGB3957, *Ruminococcaceae* SGB15119). Of note, *D.* *succinatiphilus*—the SGB with the highest likelihood to engraft (76%)—and *Phascolarctobacterium faecium* are both members of the Negativicutes class, characterized by a cell-wall composition containing lipopolysaccharides, which results in a negative Gram stain^50^. As such, these Firmicutes species may have characteristics not completely in line with those of the typical members of this phylum, possibly explaining their comparatively high engraftment rates. Among the top-engrafting non-Firmicutes species, we found several *Bacteroidales*: *Prevotella copri* clade A^51^ (strain engraftment rate = 65%), *Bacteroides finegoldii* (60%), *Bacteroides stercoris* (58%), *Alistipes putredinis* (54.2%), *Alistipes finegoldii* (53%) and *Phocaeicola massiliensis* (62%). Among Actinobacteria, the dysbiosis-associated species *Eggerthella lenta*^52^ (strain engraftment rate = 60%) and two *Bifidobacteria* (*B. bifidum* 58%, *B. longum* 55%) also exhibit high engraftment likelihood. In contrast, 19 out of the 20 least-engrafting species (strain engraftment rate < 6.5%) belonged to Firmicutes, of which 16 were members of the Clostridiales order. *Acidaminoccus intestini*, *Streptococcus salivarius* and four other unnamed and uncharacterized Firmicutes species were never found to detectably engraft in the FMT recipient despite being fairly prevalent in the donor (Fig. 3a and Supplementary Table 8). These data suggest that the engraftment potential of microbes differs among phyla and species, and that such engraftment likelihoods could be considered in future therapeutic protocols when selecting fecal donors or designing artificial microbial consortia to use instead of FMT.

We also assessed the potential transmission of eukaryotic microbes, and found that only *Blastocystis* was detectable at enough coverage to infer transmission (Methods). Most FMT screening procedures exclude donors with *Blastocystis*^3^, so its prevalence in donors is lower than in most ‘Westernized’ populations^53–55^: we detected only five donors positive for *Blastocystis* in two cohorts (BarYosephH_2020, SmillieC_2018). No transmission could be inferred (Supplementary Table 9; Methods) while two retention events in recipients were detected based on *Blastocystis* subtyping. While *Blastocystis* is increasingly reported to be linked with favorable health conditions^53,55,56^, it does not seem to play a role in FMT, possibly due to donor screening procedures, and transmission via FMT was reported as asymptomatic elsewhere^57^.

### Engraftment is linked with predicted bacterial phenotypes

We assessed whether the taxonomic differences in strain engraftment (Supplementary Table 8) we detected were associated with predicted microbial phenotypic properties. The more resistant Gram-negative species had a higher engraftment likelihood (Mann–Whitney *U*-test *Q* = 3 × 10^–6^; Supplementary Table 10), and only a few Gram-positive bacteria were among the most-engrafting species (Fig. 3a). Since most Firmicutes are Gram positive, this association may be driven by characteristics of the Firmicutes phylum unrelated to cell-wall structure. Spore-forming and motile species also tended to display reduced engraftment (Mann–Whitney *U*-test *Q* = 0.007 and *Q* = 0.008, respectively; Fig. 3a and Supplementary Table 8). All of the above suggests that species engraftment may be facilitated by specific microbial features although more refined knowledge of phenotypic traits is needed to infer mechanistic hypotheses underlying these associations.

While screening for pathogens is routinely performed as part of FMT protocols, the ability of noninfectious but disease-associated microbes to engraft remains unknown. Interestingly, microbes negatively associated with cardiometabolic health in the PREDICT 1 study^53^ tended to engraft more frequently (Spearman’s *ρ* = 0.36, *P* = 4 × 10^–7^), possibly due to more aggressive host colonization strategies or higher adaptive potential to dysbiotic or inflamed gut environments such as those found in FMT recipients. Although species prevalence in the gut of healthy individuals did not significantly correlate with engraftment across 9,120 gut metagenomic samples from 56 public studies (Supplementary Table 11; Spearman correlation, *P* > 0.05), the prevalence of bacteria in nonintestinal human body sites was associated with higher engraftment (Mann–Whitney *U*-test, *P* = 8 × 10^–4^). This suggests that ability to engraft is linked to the microbes’ capability of surviving in diverse environments. Finally, we found no association between the engraftment of individual species and clinical success (Fisher’s exact test, *Q* > 0.1; Supplementary Table 8). Together, these results show a remarkable variability in the engraftment rates among species in the human gut and suggest the possibility of screening donors to minimize the engraftment of species associated with unfavorable host conditions while promoting those with positive health associations.

### Machine learning can predict post-FMT microbial composition

**Fig. 4:**
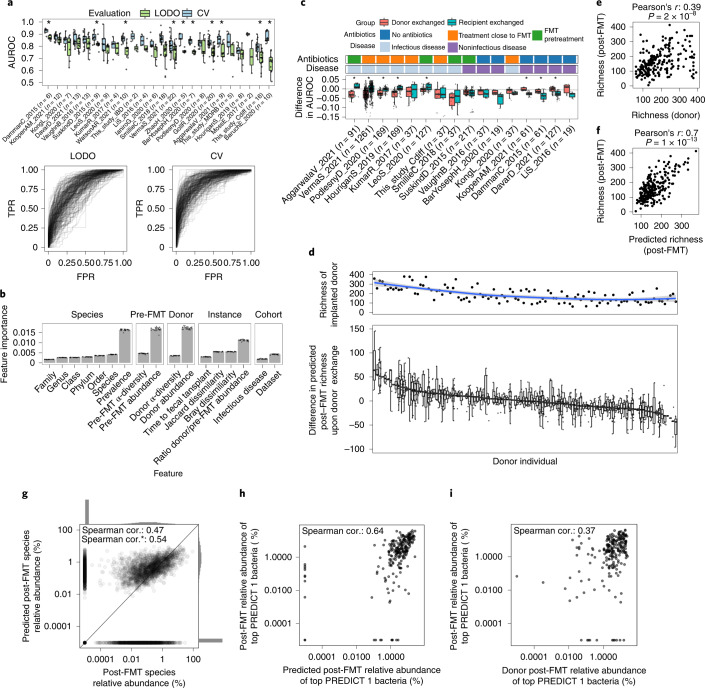
RF models predict post-FMT microbiome composition and the effect of different donors on the post-FMT microbiome. **a**, RF predictions of the presence or absence of species post-FMT. LODO and CV AUROC are reported and represented as true positive rates (TPR) versus false positive rates (FPR). **b**, The relative importance of microbial features in the LODO model (*n* = 24 for each bar). Data are presented as mean, error bars correspond to s.d. **c**, Distribution of the changes in AUROC values for the LODO models of **a** upon donor exchange (Methods). **d**, Top panel, species richness of FMT donors. The blue line is a locally estimated scatterplot smoothing fit, the shaded area corresponds to the 95% confidence interval. Bottom panel, difference in post-FMT species richness upon donor exchange with respect to the predicted post-FMT species richness of the real triad *n*(total) = 1,317. **e**, Donor species richness is positively correlated with recipient’s post-FMT species richness (Pearson’s correlation test, *r* = 0.39, *P* = 2 × 10^–8^). **f**, Predicted post-FMT species richness is strongly correlated with the actual post-FMT richness (Pearson’s correlation test, *r* = 0.7, *P* = 1 × 10^–13^). **g**, An RF regression model is able to predict bacterial abundances in the post-FMT microbiome. The asterisk designates the Spearman correlation (cor.) when omitting truly absent species predicted to be absent. Individual datasets are reported in Supplementary Fig. 10. **h**, The cumulative abundance of the top 20% PREDICT 1 bacteria post-FMT can be predicted fairly accurately using the RF regression model. **i**, Donor abundance is a worse predictor of the cumulative abundance of the top 20% PREDICT 1 bacteria than the RF regression model. Boxplots report the median and upper/lower quartiles, whiskers are at 1.5 times higher/lower of the upper/lower quartiles.

**Extended Data Fig. 6:**
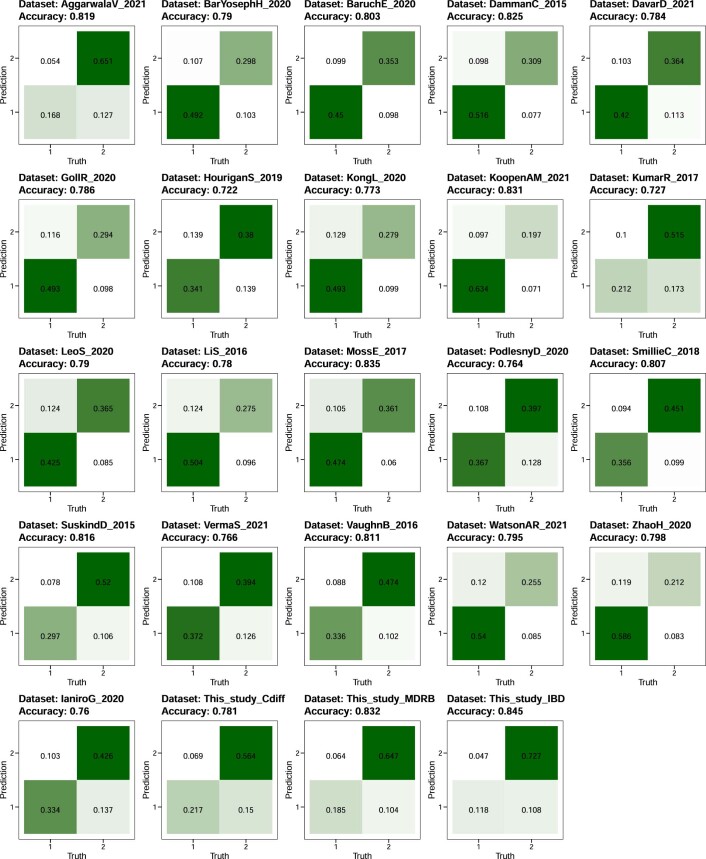
Random forest classifier prediction accuracies of post-FMT species presence/absence (CV).

Understanding what are the donor and pre-FMT microbiome factors dictating the post-FMT microbiome configuration could facilitate precision-medicine approaches for targeted microbiome modulations. Since donor strain engraftment accounts only partially for the post-FMT microbiome composition, as strains can also persist or be acquired from the environment, we developed machine learning (ML) models to predict the microbiome composition post-FMT based on a set of quantitative features. Specifically, we trained random forest (RF) models to predict the presence or absence of species post-FMT using a total of 16 microbial and host features including taxonomy, microbial abundances and *α*-diversity in pre-FMT and donor samples and microbial prevalence in unrelated cohorts (Methods). We found that these models predict post-FMT species composition with an area under the receiver operating characteristic curve (AUROC) ranging from 0.77 to 0.91 (average = 0.85, s.d. = 0.03; Extended Data Fig. 6 and Supplementary Table 12) in a fivefold cross-validation (CV) setting (Fig. 4a; Methods).

**Extended Data Fig. 7:**
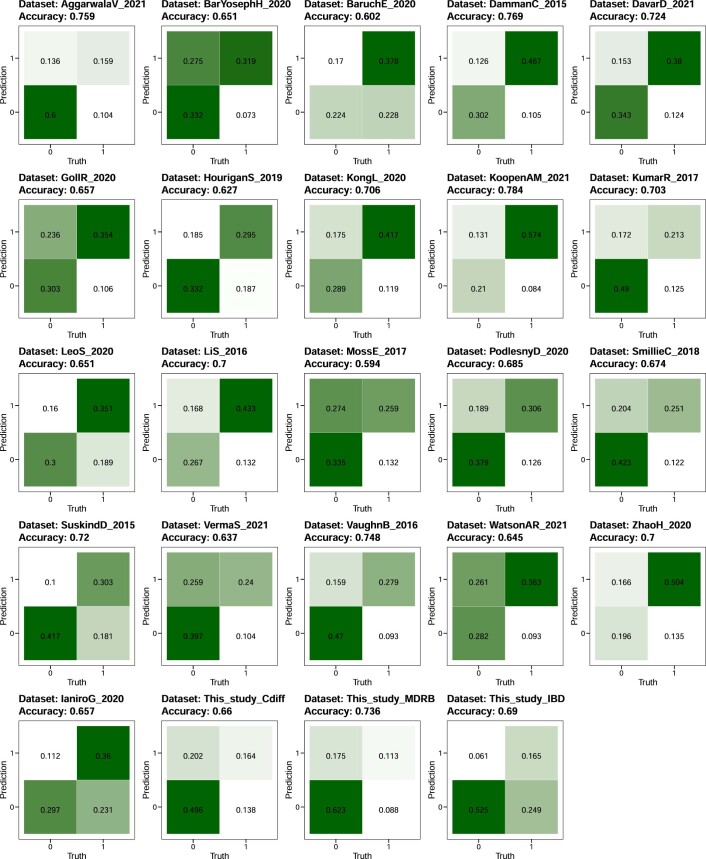
Random forest classifier prediction accuracies of post-FMT species presence/absence (LODO).

We next performed an analysis in which we predicted post-FMT species composition in a dataset by training the model on all the other datasets (leave-one-dataset-out (LODO)). In this setting, while AUROC and accuracy values were expectedly lower than in the CV setting, AUROC values were above 0.7 in all but 3 of the 24 cohorts (average = 0.77, s.d. = 0.05); Extended Data Fig. 7, Supplementary Fig. 9 and Supplementary Table 12). Finally, we evaluated RF regression models to predict the post-FMT abundance of bacterial species (Methods). These models provided estimates of the abundance of species in the post-FMT microbiome that were significantly correlated with those assessed by microbiome sequencing of the post-FMT samples (Spearman correlation 0.47, *P* < 1×10^–16^; Fig. 4g and Supplementary Fig. 10). We thus conclude that, whereas the prediction potential of the post-FMT microbiome composition is partially dependent on the cohort, substantial prediction ability is maintained across datasets.

Analysis of the importance of each feature highlighted that quantitative information on the abundance of the species in the donor and in the pre-FMT recipient as well as the overall prevalence are more relevant than characteristics such as the *α*-diversity of donor and recipient microbiomes, the *β*-diversity between donor/recipient pairs, or disease context (Fig. 4b). Single taxonomic features (that is, the species or genus labels) proved not particularly important despite differences in strain engraftment rates over different clades (Fig. 3a). This observation was, however, likely due to the effect of information redundancy and hierarchy on the importance estimates as, when we considered all taxonomic levels together, the importance of the taxonomy was comparable with that of bacterial prevalence or abundance (Supplementary Fig. 11). Further evaluation of species-wise strain engraftment rates as well as predicted microbial phenotypes (Fig. 3a and Supplementary Table 8) showed no relevant additional increase in prediction ability (mean change in CV AUROC upon addition of strain engraftment rate = –0.007, s.d. = 0.015; Supplementary Fig. 12; mean change in CV AUROC upon addition of predicted phenotypes = 0.005, s.d. = 0.019; Supplementary Fig. 13). Overall, we observed that the composition of the post-FMT microbiome is generally predictable despite differences in cohort characteristics and host conditions and the presence of a species after the transplant is dictated primarily by the amount (or absence) in the donor and in the recipient as well as taxonomy and general prevalence.

### ML models can pinpoint suitable FMT donors

**Extended Data Fig. 8:**
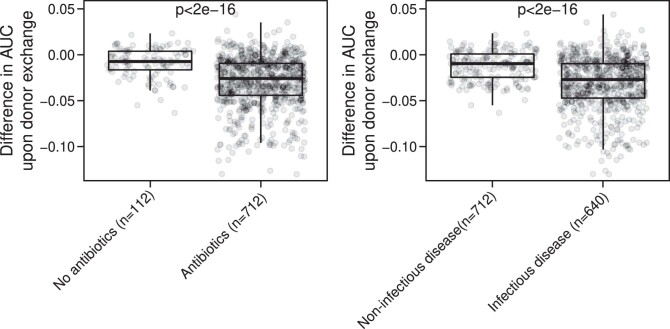
Boxplots of the difference in AUC upon simulated donor exchange. Mann-Whitney U-test two-tailed p<2e-16 for both infectious vs. non-infectious disease and antibiotics vs. no antibiotics comparisons. Box plots are defined as follows: the center line and upper and lower limit of the box correspond to the median, upper quartile and lower quartile respectively. The whiskers are defined by that data point that is at most 1.5 times higher than the upper quartile (upper whisker) or 1.5 times lower than the lower quartile (lower whisker).

To better understand to what extent the choice of the donor impacts the post-FMT gut microbiome composition, we set up a framework in which we substituted either the donor or the pre-FMT recipient of a triad with a randomly selected donor or pre-FMT recipient from a different triad of the same dataset and then evaluated the decrease in AUROC upon this exchange. We found, as expected, a decrease in predictive performance upon exchange of either donors and recipients (Fig. 4c). The performance decrease upon donor exchange was particularly pronounced in cohorts of infectious diseases and in patients pretreated with antibiotics (Mann–Whitney *U*-tests, *P* < 0.001; Extended Data Fig. 8), consistent with a higher fraction of donor strains engrafting in the recipient in these conditions (Fig. 2a). The choice of donor thus has a higher influence on the post-FMT microbiome in patients with infectious disease and/or those that were treated with antibiotics.

Finally, we investigated whether ML models can pinpoint particularly suitable donor individuals for improving microbiome features in recipients based on their individual microbiomes. We first evaluated the donor effect in modulating post-FMT species richness—a microbiome feature linked with community stability and resilience^58^ and with clinical success in the context of ulcerative colitis^5^. Upon exchange of donors in triads, we found that some donors led to a consistent increase in predicted post-FMT richness compared with the original donor, whereas others led to a decreased predicted post-FMT richness (Fig. 4d). We also found that the donors with higher richness were predicted to induce higher richness in the recipient post-FMT (Fig. 4d and Supplementary Fig. 14), and such predictions of post-FMT richness using the real donor were much more accurate than the donor’s richness alone (Pearson’s *r* = 0.7 versus *r* = 0.39, *P* = 1 × 10^–13^ versus *P* = 2 × 10^–8^; Fig. 4e,f).

**Extended Data Fig. 9:**
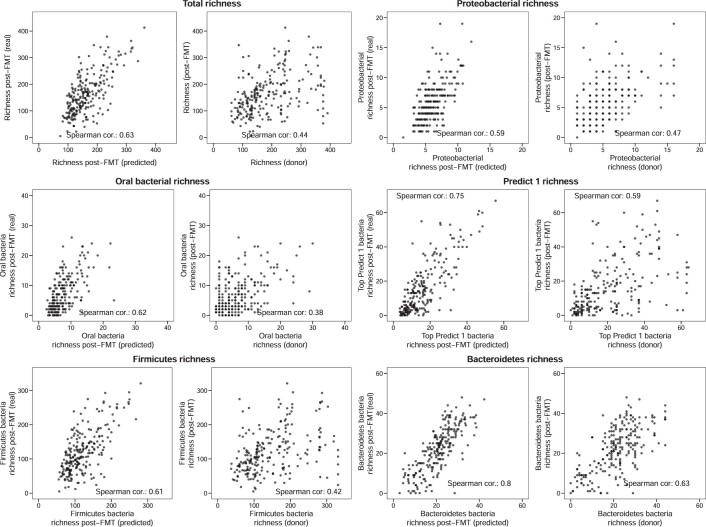
Comparisons of the predicted total species richness of bacterial groups in post-FMT samples. Predictions on the y-axis come from the RF classifier, predictions on the x-axis correspond to the cumulative richness in donor samples.

**Extended Data Fig. 10:**
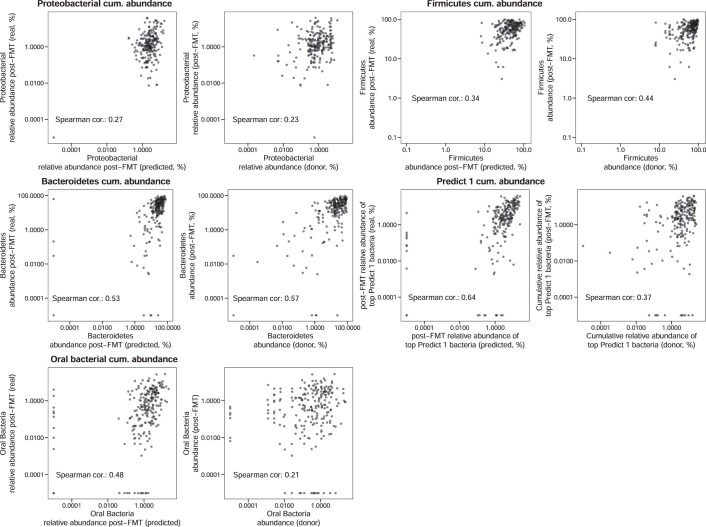
Comparisons of the predicted cumulative abundance of bacterial groups in post-FMT samples. Predictions on the y-axis come from the RF regressor, predictions on the x-axis correspond to the cumulative abundance in donor samples.

We then exploited this framework to pinpoint donors that are predicted to maximize the probability of the presence of other predefined groups of microbes in the post-FMT samples, such as Firmicutes, species found in the oral cavity (Supplementary Table 13), or the set of species found positively linked with cardiometabolic health in the PREDICT 1 study^53^ (Extended Data Fig. 9 and Supplementary Table 14). In all these situations, our models proved more accurate in predicting a given trait than using the quantitative microbial features of the donor as a direct estimator. We finally evaluated a regression model to predict the cumulative relative abundance of the same microbial groups, finding that the model can predict the cumulative abundance of microbes positively linked with cardiometabolic health better than the donor abundances alone (Fig. 4h,i), although the results are variable across different clades (Extended Data Fig. 10). Taken together, these results illustrate that our ML framework provides predictive models of the composition of the post-FMT microbial communities that might be useful for choosing a suitable donor given a specific post-FMT microbiome feature of clinical relevance, such as post-FMT microbiome richness.

## Discussion

In our meta-analysis of metagenomic samples from 24 studies investigating FMT in different diseases, we built on improved strain-level profiling approaches to assess the extent of microbial strain engraftment and retention upon FMT in relation to several clinical covariates. Donor strain engraftment varied substantially across cohorts, and such variability was explained best by mixed FMT administration routes (combining upper and lower gastrointestinal (GI) tract), by the administration in the recipient of antibiotics before FMT (therapeutically or as preconditioning), and by the recipient being affected by infectious diseases. These findings could explain the discrepancies in the effectiveness of FMT between rCDI and chronic or noninfectious disorders^4–6^. Our results provide further support for administering FMT by combined routes and including antibiotic preconditioning in FMT working protocols to increase donor microbiome engraftment, even though the potential side effects of antibiotic treatments for noninfectious diseases^59^ should be considered.

We found differential strain engraftment likelihoods associated with microbial taxonomy and phenotypic properties. Some species with immune modulation potential (for example, *Bifidobacteria* spp.), Gram-negative bacteria and some species with proinflammatory potential (for example, *Eggerthella lenta*) were more likely to engraft than most Firmicutes, including putative butyrate-producing bacteria. As FMT is performed in patients and not on healthy volunteers, it remains to be elucidated whether the general higher engraftment rates of proinflammatory microbes reflect intrinsic phenotypic traits that favor transmission and colonization in a new environment or rather a better fitness for an inflamed and dysbiotic environment. Additionally, the implementation of targeted, fine-tuned bacterial consortia as an alternative to traditional FMT would avoid the transfer of potentially detrimental bacteria (including pathogens that could remain undetected upon screening^60^), but it is still unclear whether such consortia can represent a suitable alternative to the complexity of FMT^61^.

Finally, we developed an ML model to predict the composition of the recipient’s microbiome after FMT. Given that we trained this model on different datasets and over different diseases, it performed well in comparison with a previous, single-cohort study^16^. The model we trained can predict the donors with the highest potential to shape the recipient’s microbial composition towards specific features such as increased species richness, a decreased proteobacterial richness or an increased cumulative abundance of bacteria associated with favorable cardiometabolic health. Together with a better identification of disease- and health-associated microbial features for each specific disease, this approach could lead to the development of therapeutic FMT strategies based on the selection of the recipient-specific optimal donor within a set of available donors, or the ad hoc assembly of strain consortia.

In our analysis, we integrated all available metagenomic datasets of FMT in clinical settings, but the small sample size of single studies as well as the heterogeneity of diseases and clinical protocols still prevent more clear-cut identification of predictors of post-FMT microbiome engraftment. Moreover, the link we observed between engraftment and clinical success of the FMT treatment needs to be substantiated in appropriately sized studies with higher number of patients in both outcome arms (for example, clinical failures for rCDI are relatively rare) and with more fine-grained evaluation of clinical success. Dedicated studies and randomized controlled trials are also needed to clarify the influence of protocol-related variables, such as antibiotic preconditioning or combined routes of delivery, on strain engraftment. Estimates of engraftment rates can also be refined both by sequencing samples at higher depth and by developing computational methods able to profile multiple strains from the same species co-colonizing an individual and by better accounting for nonbacterial members of the microbiome. These further improvements and investigations are needed to effectively translate the metagenomic support to FMT protocols into clinical practice.

## Methods

### Metagenomic dataset search strategy and selection

We systematically searched PubMed, Scopus and ISI Web of Knowledge as of 8 February 2021 for potentially eligible studies using the following search string: ((faecal microbiota suspension) OR (fecal microbiota suspension) OR (faecal microbiota transplant*) OR (fecal microbiota transplant*) OR (faecal microbiota donation) OR (fecal microbiota donation) OR (faecal microbiota transfer) OR (fecal microbiota transfer) OR (faecal microbiota infusion) OR (fecal microbiota infusion) OR (faecal microbial suspension) OR (fecal microbial suspension) OR (faecal microbial transplant*) OR (fecal microbial transplant*) OR (faecal microbial donation) OR (fecal microbial donation) OR (faecal microbial transfer) OR (fecal microbial transfer) OR (faecal microbial infusion) OR (fecal microbial infusion) OR (faecal suspension) OR (fecal suspension) OR (faecal transplant*) OR (fecal transplant*) OR (faecal donation) OR (fecal donation) OR (faecal transfer) OR (fecal transfer) OR (faecal infusion) OR (fecal infusion) OR (bacteriotherapy) OR (stool transplant*) OR (stool donation) OR (stool transfer) OR (stool infusion) OR (FMT)) AND ((Metagenom*) OR (shotgun) OR (engraft*) OR (whole genom*) OR (transkingdom) OR (WGS)). In addition, we manually searched the bibliographies of papers of interest to provide additional references. When needed, we contacted the authors to obtain additional data, metadata or clarification of study methods.

We considered as eligible all original studies with the following characteristics: (1) human subjects of any age were treated with nonautologous FMT; (2) shotgun metagenomic analysis of donor feces and of recipient feces (before and after treatment) was performed. We excluded studies in which the only therapeutic treatment for the disease was based on antibiotics. We further excluded those studies using microbial consortium-based transplantation approaches (instead of donor stool-based transplantations), those in which fewer than three recipients were enrolled and if raw sequencing data or metadata were not available or incomplete. In the case of randomized controlled trials that used autologous FMTs as placebo, we included only patients treated with nonautologous FMT. If studies used stool from mixed donors for FMT (multidonor FMT), they were included only if sequencing of multidonor stool batches were available. Finally, we excluded animal model studies or nonoriginal studies (reviews, meta-analyses, editorials, and so on). The eligibility of each study was assessed independently by two reviewers (N.K. and S.P.), and any disagreements were resolved by the opinion of a third reviewer (G.I.).

Sequencing data files and metadata were downloaded from public repositories as indicated in the original publications. If data were not publicly available, we contacted authors asking to provide them through private correspondence.

### Metadata extraction and curation

Metadata extraction was carried out independently by two reviewers (N.K. and S.P.), using a data collection form. Discrepancies between the two reviewers were resolved by the opinion of a third investigator (G.I.). The following data were extracted from each study if available: author names, publication year, Bioproject Accession code, sequencing depth, study location, number of total samples, study disease, number of recipients and donors, donor type (that is, whether donor individuals were related to the recipient, either family/household members or through friendship or whether they were unrelated), use of antibiotics before FMT, characteristics of infused feces (grams, volumes, use of frozen/fresh material), routes and number of infusions, follow-up, and clinical and microbiological outcomes. Data were not analyzed by sex or gender due to lack of this information in most of the published datasets.

### Newly collected metagenomic datasets

Three Italian cohorts were newly collected as case series and sequenced in the context of this study. A first cohort (This_study_Cdiff) was collected between February 2021 and August 2021 at the Fondazione Policlinico Gemelli IRCCS in Rome, Italy, and included 16 adult subjects with recurrent *C. difficile* infection and no history of other GI disorders or GI surgery. Patients were treated with a single fecal transplant from six different donors, and their stool was collected just before FMT and at different timepoints (7, 15, 30, 60, 180 and 240 days) after FMT. FMT was performed with frozen fecal material. Donor selection and manipulation of fecal material were performed following international guidelines^3^. All patients underwent FMT by colonoscopy, after bowel lavage and a 3-day vancomycin regimen, as previously described^1^. A total of 94 stool samples were sequenced. A second cohort (This_study_IBD) was collected from May 2017 to October 2017 at the Ospedale Bambino Gesù IRCCS in Rome, Italy, and included two pediatric patients with mild-to-moderately active IBD despite traditional treatments, without any active GI infection, placed central venous catheter or critical illness or comorbidity. They received a single FMT (one patient from a related donor, the other from an unrelated donor). Stool samples were collected and sequenced at follow-up visits up to 30 days after treatment, yielding eight metagenomic samples. A third cohort (This_study_MDRB), from the Ospedale Pediatrico Bambino Gesù IRCCS in Rome, Italy, included, between October 2018 and March 2019, five pediatric patients with large bowel colonization with MDRB and either acute leukemia (*n* = 4 patients) or severe combined immunodeficiency (*n* = 1 subject). Patients underwent single (*n* = 4 subjects) or sequential (*n* = 1 subjects, *n* = 2 procedures) fecal transplant from one of two donors. Stool samples were collected and sequenced at follow-up visits up to 30 days after FMT (*n* = 13 metagenomic samples in total). In both pediatric cohorts, FMT was performed as previously described^63^. Written informed consent was obtained from all participants (or the parents of pediatric participants). No compensation was provided to the participants. Consistent metadata of all 115 samples newly collected in this study can be found in Supplementary Table 2.

Samples were collected using a stool collector with a DNA stabilization buffer, brought directly by patients to the FMT centers in a refrigerated box within 6 h from collection, and then stored at –80 °C for up to 36 months before being shipped in dry ice to the CIBIO Department (Trento, Italy) for DNA extraction and sequencing. DNA extraction was performed using the DNeasy PowerSoil Pro Kit (Qiagen) according to the manufacturer’s procedures. No human DNA sequence depletion or enrichment of microbial or viral DNA was performed. DNA concentration was measured with Qubit (Thermo Fisher Scientific) and DNA was then stored at –20 °C. Sequencing libraries were prepared using the Illumina DNA Prep (M) Tagmentation kit (Illumina) following the manufacturer’s guidelines. Sequencing was performed on the Illumina NovaSeq 6000 platform at a target sequencing depth of 7.5 Gbp following the manufacturer’s protocols.

Newly generated shotgun metagenomic sequences were preprocessed and quality controlled using the pipeline available at https://github.com/SegataLab/preprocessing and KneadData within bioBakery v.3 (ref. ^23^). Shortly, reads were quality controlled and those of low quality (average quality score <Q20), fragmented (<75 bp) and with more than two ambiguous nucleotides were removed with Trim Galore (v.0.6.6). Contaminant and host DNA was identified with Bowtie2 (v.2.3.4.3)^64^ using the parameter ‘-sensitive-local,’ allowing confident removal of the phiX 174 Illumina spike-in and human reads (hg19 human genome release). Remaining high-quality reads were sorted and split to create forward, reverse and unpaired reads output files for each metagenome. Average sequencing depth after preprocessing was 7.3 s.d. 4.9 Gbp. Sequencing depth of each sample can be found in Supplementary Table 2.

### Definition of clinical response across studies

To evaluate the association between microbial engraftment and clinical success, we identified all studies that expressed clinical outcomes as binary variables, for which single individual metadata were available or could be retrieved from the publication via manual curation, and for which both the clinically successful and the unsuccessful groups had at least one FMT triad. Ten published studies (AggarwalaV_2021, BarYoseph_2020, BaruchE_2020, DavarD_2021, GollR_2020, SmillieC_2018, SuskindD_2015, VaughnB_2016, ZhaoH_2020, IaniroG_2020) and the three new cohorts (This_Study_Cdiff, This_Study_IBD, This_Study_MDRB) were included. Clinical success was defined as *C.* *difficile* infection cure in three studies (AggarwalaV_2021, SmillieC_2018, This_Study_Cdiff), as eradication of MDRB in two studies (BarYoseph_2020, This_Study_MDRB), as objective tumor regression by imaging according to iRECIST criteria^65^ in two studies (BaruchE_2020, DavarD_2021), as reduction by more than 75 points in the IBS-Severity Scoring System (IBS-SSS) in GollR_2020, as resolution of diarrhea in IaniroG_2020, as reduction by >25% in the Yale Global Tic Severity Scale (YGTSS-TTS) and reduction by more than three in the Harvey-Bradshaw Index (HBI) change without an increase in IBD-related medications in VaughnB_2016, as clinical remission expressed as Pediatric Crohn’s Disease Activity Index (PCDAI) of less than ten in SuskindD_2015, and as clinical remission expressed as Pediatric Ulcerative Colitis Activity Index (PUCAI) of less than ten in This_Study_IBD.

### Building the expanded SGB database

SGBs are clusters of microbial genomes and MAGs defined to have no more than 5% pairwise genetic divergence^25^. SGBs can contain taxonomically labeled microbial genomes from isolate sequencing (kSGBs) or can lack taxonomic contextualization from isolate sequencing (uSGBs; that is, SGBs with no cultured isolate). In this work, we first extended the SGB database and then employed it to detect and profile the taxa present in metagenomes belonging to any kSGB or uSGB at species- and strain-level resolution.

The custom extended database was built starting from the 154,723 MAGs and 80,990 reference isolate genomes from Pasolli et al.^25^ and further expanded using the same approach with 616,805 MAGs from different human body sites, animal hosts and other environments, together with 155,767 reference genomes in the National Center for Biotechnology Information GenBank database^66^ available as of November 2020. MAGs were assembled from metagenomes by applying metaSPAdes^67^ (v.3.10.1) or MEGAHIT^68^ (v.1.1.1) to each sample separately as reported in Pasolli et al.^25^. Obtained assembled contigs longer than 1,500 nucleotides were binned into MAGs with MetaBAT2 (ref. ^69^) (v.2.12.1). We executed CheckM (v.1.1.4)^70^ on the 1,008,148 genomes, filtering those with completeness below 50% or contamination above 5% to ensure high quality. Next, we minimized the redundancy among genomes by computing Mash distances^71^ on the quality-controlled sequences, and dereplicating sequences at 99.99% genetic identity. A total of 729,195 genomes (560,076 MAGs (Supplementary Table 15) and 169,119 reference genomes) were kept in the extended database used for species- and strain-level profiling, thus leveraging reference-based profiling with information provided by metagenome assembly. Reference isolate genomes and MAGs were then clustered into SGBs spanning at least 5% genetic diversity, and SGBs to genus-level genome bins (GGBs; 15% genetic diversity) and family-level genome bins (FGBs; 30% genetic diversity), following the procedure described in Pasolli et al.^25^. ‘phylophlan_metagenomic’—a subroutine of PhyloPhlAn 3^72^ that applies Mash^71^ to estimate the whole-genome average nucleotide identity among genomes—was used to assign MAGs to SGBs. Reference genomes and MAGs for which no SGB with at least 5% average genetic distance was present in the database were assigned to new SGBs based on the average linkage hierarchical clustering (with the dendrogram cut at 5% genetic distance). Similarly, when no GGBs or FGBs below the genetic distance threshold existed, SGBs were assigned to new GGBs and FGBs following the same procedure.

Prokka (v.1.12 and v.1.13)^73^ was used to annotate the open reading frames of all reference genomes and MAGs. Coding sequences were assigned to a UniRef90 cluster^74^ by performing a Diamond search (v.0.9.24)^75^ of the coding sequences on the UniRef90 database (v.201906) and assigning a UniRef90 identifier when the mean sequence identity to the centroid sequence was greater than 90% and covered more than 80% of the centroid sequence. Sequences that could not be assigned to any UniRef90 cluster following this procedure were de novo clustered with MMseqs2 (ref. ^76^) to SGBs following the Uniclust90 criteria^77^.

### Definition of kSGBs and uSGBs and taxonomic assignment

SGBs containing at least one reference genome (kSGBs) were assigned the same species-level taxonomy of the reference genomes included in the kSGB following a majority rule. SGBs containing no reference genomes (uSGBs) were given the taxonomic annotation of the corresponding GGB (up to the genus level) if this included reference genomes, and of the FGB (up to the family level) if that included reference genomes. Alternatively, if no reference genomes were contained in the FGB, a phylum-level taxonomic label was assigned based on the majority rule of up to 100 closest reference genomes to the MAGs in the SGB as determined by ‘phylophlan_metagenomic’. Taxonomic assignment of SGBs profiled in this study can be found in Supplementary Table 3.

### Species-level profiling of metagenomic samples

Species-level profiling was performed on samples sequenced to a depth higher than 1 Gbp (*n* = 1,419; 100 samples being excluded from downstream analyses) using MetaPhlAn 4 (ref. ^23,39﻿^)﻿ with default parameters and the custom extended SGB database. uSGBs with fewer than five MAGs were discarded, as there is a higher risk of them being the result of assembly artifacts or chimeric sequences. Next, SGB core genes were defined as ORFs in a UniRef90 family or in a de novo clustered gene family (based on the Uniclust90 clustering procedure^77^) that were detected in at least half of the genomes of the SGB. Core genes were further filtered by selecting the highest threshold that allowed obtaining at least 800 core genes. The obtained core genes were then split into fragments of 150 nt, and such fragments were then aligned against the genomes of all SGBs using Bowtie2 (v.2.3.5.1; –sensitive option)^64^. Marker genes of a SGB were defined as core genes whose fragments were found in less than 1% of the genomes of any other SGB. When fewer than ten marker genes were found for a SGB, conflicts were defined as occurrences of more than 200 of its core genes in more than 1% of the genomes of another SGB. All conflicts for each SGB were then retrieved to generate conflict graphs. Conflict graphs were processed iteratively, and SGBs were merged for each conflict to both minimize the number of merged SGBs and maximize the number of markers. Finally, a maximum of 200 marker genes were selected for each SGB, prioritizing first their uniqueness and next the larger sizes. SGBs with fewer than ten markers were discarded at this point. Merged SGBs (*SGB_group*) profiled in this study can be found in Supplementary Table 3. The resulting 5.1 M marker genes (average: 189 ± 34.25 s.d. marker genes/SGB) were used as a new reference database for MetaPhlAn 4 (species-level profiling) and StrainPhlAn 4 (strain-level profiling). The presence of *Blastocystis* and the identification of its different subtypes was inferred with a mapping-based computational pipeline described elsewhere^55^.

### Strain-level profiling of metagenomic samples

Strain profiling was performed with a modified version of StrainPhlAn 3 (ref. ^23^) using the custom SGB marker database described above that has been released as StrainPhlAn 4^39^. We modified the StrainPhlAn code to change the sample and marker filtering behavior to allow for profiling more samples and SGBs. A sample was kept as long as it had at least 20 markers (parameter–sample_with_n_markers) and a marker was kept as long as it was present in 50% of the samples (parameter–marker_in_n_samples). After this first filtering, we retained samples with at least ten markers (parameter–sample_with_n_markers_after_filt). All 2,576 SGBs profiled by MetaPhlAn were initially considered for the strain-level profiling.

To improve accuracy of strain sharing detection and to more confidently define strain identity, we additionally considered samples from curatedMetagenomicData (cMD) R package^78^ (v.3.15). We included 4,443 human gut metagenomic samples from 962 individuals older than 6 years from ‘Westernized’ populations (as defined in cMD) that were sampled longitudinally, obtained from 18 datasets (Supplementary Table 11). For each subject and each SGB, two samples being at most 6 months apart were selected. When more than two timepoints close in time were available, we selected the pair that maximized the lower estimated coverage of the SGB among the two samples, that is, maximized their chance to pass the filtering steps in StrainPhlAn. In case of ties, we took those with higher coverage. Coverage of an SGB in a sample was estimated as [sample sequencing depth] × [relative abundance of the SGB] / [estimated genome length], with estimated genome length being extracted from the MetaPhlAn enlarged database described above. For kSGBs this is determined using only the genome lengths of the reference genomes in the kSGB, whereas for uSGBs 7% is added to the average genome length (estimated to be the average difference between the genome sizes of reference genomes and MAGs within the same SGB).

We included in the strain analysis samples as primary (that is, those that are used to select markers, parameter–samples) if they had an estimated coverage of at least 2X that of a given SGB genome, otherwise they were added as secondary samples (that is, those that are added only after the markers are selected with the primary samples, parameter–secondary_samples). In total, 1,033 SGBs that were detected in at least 20 primary samples were profiled at the strain level. To exclude strains likely coming from food sources, we included 216 MAGs in 19 SGBs (Supplementary Table 16) coming from food samples^79^ and used them in the StrainPhlAn profiling with the –secondary_references parameters. Samples that had StrainPhlAn mutation rates less than 0.0015 to any food MAG were discarded following the same procedure as in (Valles-Colomer et al., manuscript in preparation). SGBs in which more than 20% of the samples would be discarded using this criterion—constituting in large part of strains regularly found in food—were fully excluded (*n* = 3 SGBs: *Bifidobacterium animalis* SGB17278, *Lactobacillus acidophilus* SGB7044, *Streptococcus thermophilus* SGB8002). Additionally, we excluded 7 SGBs for which the marker genes alignment length was shorter than 1,000 nucleotides, and another 11 SGBs for which StrainPhlAn was not successful in building a phylogenetic tree.

### Inference of strain transmission events

We obtained phylogenetic distances between strains as their leaf-to-leaf branch lengths along the trees (that is, patristic distances) produced by StrainPhlAn (built on marker genes alignments, retaining positions with at least 1% variability), normalized by dividing them by the median phylogenetic distance. As no consensus definition of strain is currently available, to infer strain identity and supported by the clear bimodal distribution of patristic distances of strains from the same individual with the highest peak in 0 (ref. ^22^), we defined and applied operational species-specific definitions by identifying the threshold that optimally separated phylogenetic distance distributions of strains of a given species in the same individual sampled at two timepoints (same strain), to that in unrelated individuals (different strains) whenever enough data were available. For all strain-level profiled SGBs, we determined the phylogenetic distance threshold that best separates strains from the same subject (different post-FMT timepoints of the same recipient or different samples of the same donor subject or different additional longitudinal samples of the same subject, always less than 6 months apart) from those of unrelated subjects with no possibility of direct transmission (subjects in different datasets) in the datasets we used in this study. For SGBs for which at least 50 same-individual and 50 unrelated comparisons were available, we determined the threshold that maximizes Youden’s index (defined as sensitivity + specificity – 1). If the resulting calculated threshold was greater than the fifth percentile of the distribution of subjects in different datasets, we adjusted the threshold to the 5th percentile as a bound on the false discovery rate (FDR). For SGBs for which fewer than 50 same-individual comparisons but at least 50 unrelated comparisons were available (in which optimal thresholds cannot reliably be estimated), we used the third percentile of the interindividual phylogenetic distances of subjects in different datasets, which corresponded to the median of all the calculated percentiles in (Valles-Colomer et al., manuscript in preparation). SGBs for which fewer than 50 unrelated comparisons were available (*n* = 17) were discarded. The SGB-specific phylogenetic distance thresholds for all 995 strain-level analyzed SGBs can be found in Supplementary Table 3. Finally, we defined strain identity for pairs of strains when their pairwise genetic distance fell below the SGB-specific thresholds.

### Sample filtering

Strain-level profiling allows identification of mislabeled samples^80^. We identified and excluded post-FMT samples (*n* = 21 out of 1,419) that did not share any strain with neither their corresponding pre-FMT sample nor the donor’s sample—something highly unexpected due to the high temporal stability of the gut microbiome^22,23,36,81^ and thus potential cases of sample mislabeling. We also identified outliers with more than 20 shared strains between pre-FMT and donor samples while being from two supposedly unrelated individuals (*n* = 2 cases; Supplementary Fig. 15), most probably not representing true recipient–donor pairs. The third outlier with more than 20 shared strains was coming from a dataset using both related and unrelated donors, but the Bray–Curtis dissimilarity between the donor and pre-FMT samples was close to zero (Bray–Curtis = 0.019) suggesting they are the same biological sample and confirming the mislabeling. Finally, we excluded the ZouM_2019 cohort from the analysis because strain-sharing sample clustering was heavily discordant from the grouping of FMT triads according to the metadata (Extended Data Fig. 1) and ZouM_2019 was the only dataset with a median of only one strain shared between post-FMT and donor samples (Supplementary Fig. 16), further suggesting systematic errors in the metadata.

### Inferring donor subject grouping

In three cohorts (BarYosephH_2020, DammanC_2015 and LeoS_2020) some donors provided stool material to multiple recipients, but we could not solve which donor samples were transferred to which patients, either from the metadata or through private correspondence with the authors. Therefore, we inferred grouping of donor samples into subjects using strain sharing: donor samples sharing more than 15 strains were grouped into one subject. This threshold allows confident matching of samples from the same subject, since unrelated samples very rarely share more than five strains (0.08% of pairs of samples), whereas longitudinal post-FMT samples frequently share more than 15 (56.8% of pairs of samples; Supplementary Fig. 17) as also reported elsewhere^22^. Indeed, in these three datasets samples from the same assigned donor always shared at least 15 strains, while this was never observed among samples from different donor individuals.

### Inferring donor–recipient matching

Donor–recipient matching was unavailable for DammanC_2015 and we were unable to obtain it through private correspondence with the authors. However, as at least one post-FMT sample of a recipient always shared eight or more strains with one donor subject, while no post-FMT samples of the same recipient shared eight or more strains with any other donor subject (Supplementary Fig. 18), we used the criterion of sharing eight or more strains to infer donor–recipient matching in the dataset.

### Definition of FMT triads

We considered only complete FMT triads, that is, sets of at least one sample from the recipient pre-FMT, at least one from the donor, and at least one from the recipient post-FMT. In case of multiple sequential FMT transplants, we included only the first one. In case of multiple pre-FMT samples, we used the one collected closest to the FMT. When multiple donor samples were available and there was no indication of which one was used, we picked one randomly since donor samples from the same individual are reasonably stable in terms of species-level composition and strain identity^8,22^ (Supplementary Fig. 19). Finally, when multiple post-FMT samples were available, we picked the one closest to 30 days post-FMT, which is the value that minimizes the sum of absolute deviations of timepoints (Supplementary Fig. 1). Where there was more than one round of treatment, we considered only those post-FMT samples that were taken before the second treatment round.

### Assessing strain sharing, retention and engraftment

We defined strain-sharing rates as the total number of shared strains between two samples divided by the number of species profiled by StrainPhlAn in common between the two samples. To quantify the fraction of post-FMT strains that were already present pre-FMT or that are shared with the donor, we defined the fraction of retained strains as the fraction of post-FMT strains shared with pre-FMT (shared strains between post-FMT and pre-FMT divided by the number of strains profiled at post-FMT) and the fraction of donor strains as the fraction of post-FMT strains shared with the donor (shared strains between post-FMT and donor divided by the number of strains profiled at post-FMT).

Next, we determined the number of engrafted strains as the (absolute) number of shared strains between post-FMT and the donor excluding the strains shared between pre-FMT and the donor samples. In this context we defined four categories that describe the relationship between donor- and recipient individuals (Fig. 1e). ‘Related’: individuals are genetically related or cohabiting/friends; ‘unrelated’: individuals are neither genetically related nor cohabiting/friends as stated in the study manuscript, recruited through public advertisement or hospital’s cohorts; ‘mixed’: only some of the individuals are genetically related or cohabiting/friends; ‘unknown’: the relation of donors to recipients was not stated in the manuscript or metadata. The number of strains that could engraft is defined as the number of cases in which StrainPhlAn can profile the strain in the donor sample while excluding both the shared strains between pre-FMT and donor and the cases where the species is present in the post-FMT, but no strain is profiled by StrainPhlAn (as in these cases it is not possible to determine the strain identity). Finally the strain engraftment rate was defined as the number of engrafted strains divided by the number of strains that could engraft. This measure was computed for each FMT triad (by aggregating over species) and also for each species (by aggregating over FMT triads). In the latter case, only species with at least 15 FMT triads from at least four datasets in which the strain could engraft were included in the analyses.

### Visualization and ordinations of strain sharing in cohorts

To visualize strain sharing in datasets, we computed networks as well as t-SNE plots based on the number of shared strains between pairs of samples. Unsupervised networks were visualized using the igraph package in R (v.1.2.6)^82^ with the Fruchterman–Reingold layout algorithm with squared edge weights, with edges being the number of shared strains and nodes representing samples. Only edges with more than one shared strain are shown. The t-SNE plot was generated using the scikit-learn package^83^ in Python (v.1.0.2) with perplexity set to 20 and remaining parameters left default.

### Comparing strain- and species-level *β*-diversities for FMT triad clustering

To compare how well strain- and species-level information allow clustering of samples from the same FMT triads, we performed K-medoids clustering with partitioning around medoids (PAM) algorithm implemented in scikit-learn-extra Python package (v.0.2.0) using strain sharing rates dissimilarities (defined as 1 – strain sharing rate) as compared with Aitchison distance and Bray–Curtis dissimilarity (on untransformed data, after arcsine square root transformation and after logit transformation). In case of Aitchison distance, the zeros were replaced by the per taxon minimal nonzero abundance and in case of logit transformation the zeros were replaced by the half of the minimal nonzero abundance globally. Clustering quality was assessed using the clustering purity, which is defined as the fraction of samples that belong to the majority class in their respective cluster. When calculating the purity of FMT triads with shared donor samples (donor samples having been administered to several recipients), we treated the single sample as multiple samples, each belonging to one of the associated FMT triads. In this way the association was considered pure if the donor sample was clustered with any of the triads it belongs to.

### Prevalence of the SGBs across different human body sites

We profiled 9,900 healthy human microbiome samples from 59 datasets spanning different body sites (airways, gastrointestinal tract, oral, skin and urogenital tract; Supplementary Table 11) using MetaPhlAn 4 (ref. ^23,39^) with default parameters and the custom SGB database (see above). Only individuals older than 3 years and from cohorts involving industrialized nonrural populations (defined as ‘Westernized’ in cMD^78^) were considered. Age, lifestyle and disease status were considered as reported in cMD^78^.

### Annotation of SGB phenotypic traits

SGB phenotypes were predicted using Traitar (v.1.1.12)^62^ on the genes present in 50% of genomes available for each SGB in the custom SGB database. Only annotations for which the *phypat* and the *phypat* + *PGL* classifiers predictions were in agreement were used.

### Statistical analysis

Total strain-sharing variance explained by FMT triad membership (Fig. 1a) was assessed by PERMANOVA on strain-sharing-based dissimilarities using the adonis function in the vegan package in R (v.2.5–7)^84^. Dissimilarities were computed within each dataset as 1 – (*n*/*M*), where *n* is the number of shared strains and *M* is the maximum of the number of shared strains.

To compare differences between median strain sharing or engraftment measures (Figs. 1e and 2a,b) in two groups of datasets against the null distribution, permutation tests were applied by randomly permuting the assignments between labels and dataset identifiers 9,999 times.

LOESS fit in Fig. 4d was computed using the geom_smooth function from the ggplot2 (v.3.3.5) in R with standard parameters.

To compare median strain-sharing rates between triads in which the FMT procedure was clinically defined as ‘successful’ and those in which was clinically ‘unsuccessful’ (see above) (Fig. 2c), we applied four statistical tests. First, we used a permutation test applied by randomly permuting the success labels within each dataset 9,999 times. Second, we fitted a linear mixed model predicting strain engraftment rate with the clinical success as an indicator variable and the dataset identifier as a random effect using the R package lme4 (ref. ^85^); the significance was assessed by performing a likelihood-ratio test against a null model without the success indicator variable. Third, we computed median strain sharing rates of successful and unsuccessful groups within each dataset and compared the medians of the successful group with the unsuccessful groups with the Wilcoxon signed-rank test as implemented in the SciPy package^86^ (v.1.7.3) in Python. Correction for multiple testing (Benjamini–Hochberg procedure, *Q*) was applied when appropriate with significance defined at *Q* < 0.1.

### Multivariate analysis

A multivariate analysis was performed to assess associations between strain engraftment rates and clinical/nonclinical variables. We included both covariates describing the clinical process, the recipient’s and donor’s microbiomes, and experimental variables consistently available across studies: antibiotics intake (that is, intake close to FMT treatment, intake as a FMT pretreatment or no antibiotic intake); whether the FMT was done to treat an infectious or noninfectious disease; administration of fresh or frozen stool; the amount of feces administered (in grams); the route of FMT administration categorized in ‘upper GI’ routes (capsules, enteroscopy, nasogastric tube, nasoduodenal tube, upper endoscopy, PEG), ‘lower GI’ routes (colonoscopy) and ‘mixed’ routes (FMT protocols utilizing both upper and lower routes for the same recipient); recipient’s age (in years); recipient’s and donor’s *α*-diversity (Shannon index on species-level abundances); the Bray–Curtis *β*-diversity and strain-sharing rate between recipient pre-FMT and donor; usage of bead-beating steps for DNA extraction; broad geographic regions based on the recipient’s lifestyle and diet (Mediterranean consisting of Israel, Italy and France^87^; North America consisting of the United States and Canada; Central and Northern Europe consisting of Norway, the Netherlands and Germany; and China). Categorical variables were converted to sets of binary variables, one per each category level (one-hot encoding). All variables were standardized by subtracting the mean and dividing by the s.d.

Since many variables in the analysis are correlated with each other (Supplementary Fig. 6), we performed partial least squares decomposition, which is well-suited for multicollinear data, where the standard linear models are inappropriate. We used the PLSRegression class with parameter scale=False from the scikit-learn^83^ Python library (v.1.0.2). The coefficients for each variable composing each component were retrieved through the x_weights_ parameter and the transformed data matrix through the x_scores_ variable returned from the fit_transform method. We regressed each component separately on the strain engraftment rate with ordinary least squares. The first two components were explaining the most the strain engraftment rate and were the only ones significantly associated with it (*R*^2^ = 0.187, *Q* = 6 × 10^–10^ and *R*^2^ = 0.046, *Q* = 3.8 × 10^–3^ for the first and second component, respectively; Extended Data Fig. 5). We assessed the association of the variables with the components by hierarchical bootstrap, that is, by resampling the datasets and for each dataset resampling the FMT triads and the associated variables. By resampling the data matrix this way and repeating the PLS decomposition (9,999 iterations) we obtained an estimate of empirical distribution for each weight coefficient.

### Machine learning

We used an ML modeling approach to predict the taxonomic composition (presence/absence and relative abundance) of the post-FMT microbiome. To this end, we first organized the data such that each datapoint represented a species in a specific FMT triad. We did not consider species absent in both recipient pre-FMT and donor. As features associated with each datapoint we used information specific to each FMT triad (Jaccard distances and Bray–Curtis dissimilarities between pre-FMT and donor samples as estimates for their microbiome compositional similarity, ratio of pre-FMT and donor species abundances, time between FMT and sample collection), species relative abundances for all samples (abundances in the post-FMT were treated as the dependent variables), and Shannon entropy values for pre-FMT and donor samples, information about species (taxonomy, prevalence in an unrelated set of metagenomic samples^23^) and cohort-specific information (dataset, disease infectivity).

We trained RF models^88^ both in a LODO as well as in a fivefold CV fashion. In the CV setting, we repeated the entire training/evaluation with five resamplings and averaged the prediction probabilities. To avoid overestimating model performance, we omitted species that were absent in both pre-FMT and donor samples in the evaluation step since those are easy to predict (Fig. 4a,b). Training and evaluation of RF models was done using the classif.ranger learner (for the presence/absence classifier) and regr.ranger (for the relative abundance regressor) from the mlr3 package (v.0.10) in R^89^ with parameter importance = ‘permutation’. We used the unbiased AUROC metric to evaluate the performance of the presence/absence classifier. Feature importance values were obtained directly from the trained RF regression model. Reported AUROC values were calculated per FMT triad and correspond to the AUROC of the predicted post-FMT species against the species actually detected in the post-FMT sample.

The pre-FMT/donor exchange simulations are based on the idea that we can exchange the real pre-FMT/donor individuals with others (from different FMT triads) in silico and then predict and analyze the post-FMT microbiome of these artificial triads. (Fig. 4c,d). Here, we chose random pre-FMT/donor samples from a different FMT triad of the same dataset and exchanged all associated features. We ensured that donor samples came from a different FMT triad and from a different donor individual (since some donor individuals donated stool to more than one FMT triad). In these experiments, we only considered datasets with at least three donors.

To evaluate the ability of the presence/absence classifier to predict continuous post-FMT microbiome traits (Fig. 4e,f,h,i), we computed the predicted species richness of certain groups of bacteria (richness, proteobacterial richness, Firmicutes richness, Bacteroidetes richness, PREDICT 1 species richness (Supplementary Table 14), richness of oral bacterial (Supplementary Table 13). We summed up raw prediction probabilities to estimate richness values. Similarly, for the evaluation of the abundance regressor, we computed the predicted cumulative abundance of the same groups of bacteria described above.

### Reporting summary

Further information on research design is available in the Nature Research Reporting Summary linked to this article.

## Online content

Any methods, additional references, Nature Research reporting summaries, source data, extended data, supplementary information, acknowledgements, peer review information; details of author contributions and competing interests; and statements of data and code availability are available at 10.1038/s41591-022-01964-3.

## Supplementary information

Supplementary Information

Reporting Summary

Supplementary Tables Supplementary Tables 1–16.

## Data Availability

Newly generated shotgun metagenomics sequencing data are available at the European Nucleotide Archive under accession number PRJEB47909. Metadata are available in Supplementary Table 2 and in curatedMetagenomicData^78^.

## Code availability

StrainPhlAn 4 was used for strain-sharing inference, and is available at https://github.com/biobakery/MetaPhlAn. The code to reproduce the ML results can be found under the following link: http://segatalab.cibio.unitn.it/data/FMT_meta.html. All analyses were performed using open-source software.
