# Supplementary material for: Variability of strain engraftment and predictability of microbiome composition after fecal microbiota transplantation across different diseases
Source: Nat Med. 2022 Sep 15;28(9):1913–23. doi: 10.1038/s41591-022-01964-3 (PMC9499858; doi:10.1038/s41591-022-01964-3)
Supplement: Supplementary file 1 — Supplementary Information [file 41591_2022_1964_MOESM1_ESM.pdf]

nature portfolio

https://doi.org/10.1038/s41591-022-01964-3

Supplementary information

Variability of strain engraftment and predictability of microbiome composition after fecal microbiota transplantation across different diseases

In the format provided by the authors and unedited

Variability of strain engraftment and predictability of microbiome composition after fecal microbiota transplantation across different diseases

Gianluca Ianiro*[,1,2], Michal Punčochář*[,3], Nicolai Karcher*[,3], Serena Porcari [1,2], Federica Armanini [3], Francesco Asnicar [3], Francesco Beghini [3], Aitor Blanco-Míguez [3], Fabio Cumbo [3], Paolo Manghi [3], Federica Pinto [3], Luca Masucci [4,5], Gianluca Quaranta [4,5], Silvia De Giorgi [1,2], Giusi Desirè Sciumè [1,2], Stefano Bibbò [1,2], Federica Del Chierico [6], Lorenza Putignani [6], Maurizio Sanguinetti [4,5], Antonio Gasbarrini [1,2], Mireia Valles-Colomer^[,3], Giovanni Cammarota^[,1,2], Nicola Segata^[,3,7]

1. Digestive Disease Center, Fondazione Policlinico Universitario "A. Gemelli" IRCCS, Rome, Italy
2. Department of Translational Medicine and Surgery, Catholic University of Rome, Rome, Italy
3. Department CIBIO, University of Trento, Trento, Italy
4. Microbiology Unit, Fondazione Policlinico Universitario "A. Gemelli" IRCCS, Rome, Italy
5. Department of Basic Biotechnological Sciences, Intensivological and Perioperative Clinics, Catholic University of Rome, Rome, Italy
6. Department of Diagnostic and Laboratory Medicine, Unit of Parasitology and Multimodal Laboratory Medicine Research Area, Unit of Human Microbiome, Bambino Gesù Children's Hospital IRCCS, Rome, Italy
7. IEO, Istituto Europeo di Oncologia IRCSS, Milan, Italy

* These authors contributed equally.

^ These authors jointly supervised this work.

Corresponding authors: Gianluca Ianiro gianluca.ianiro@unicatt.it, Nicola Segata nicola.segata@unitn.it

EXTENDED DATA FIGURES

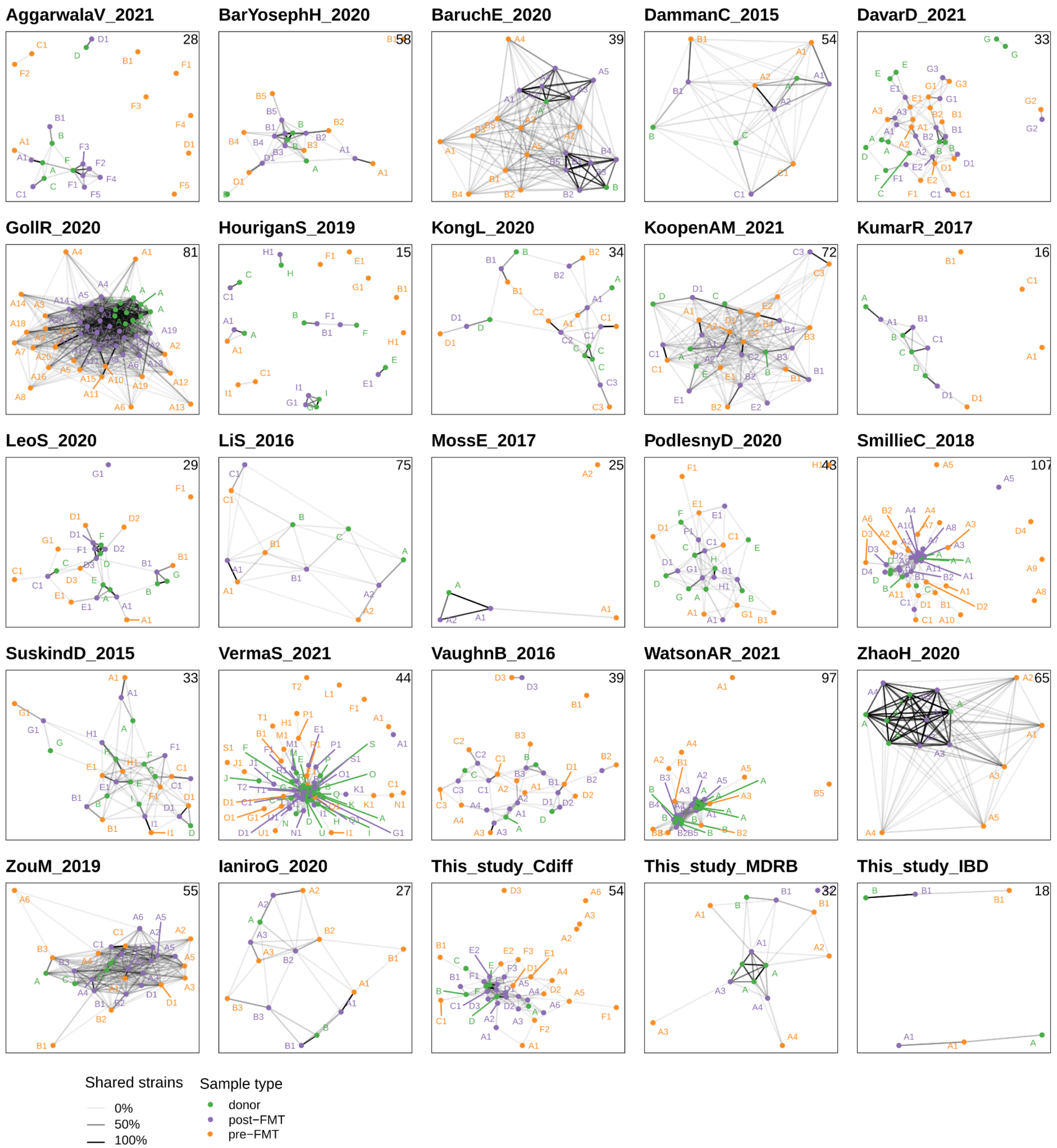

ED Figure 1. Strain sharing networks for the datasets included in this study not shown in Fig. 1A. Each node corresponds to a sample and is colored by its role in FMT triads (recipient pre-FMT sample, recipient post-FMT sample, and donor's sample). Edge opacity is proportional to the number of shared strains between two samples (*Methods)* and only edges corresponding to at least 2 shared strains are shown. The structure of the networks illustrates how FMT triads tend to cluster together but with different clustering characteristics across cohorts.

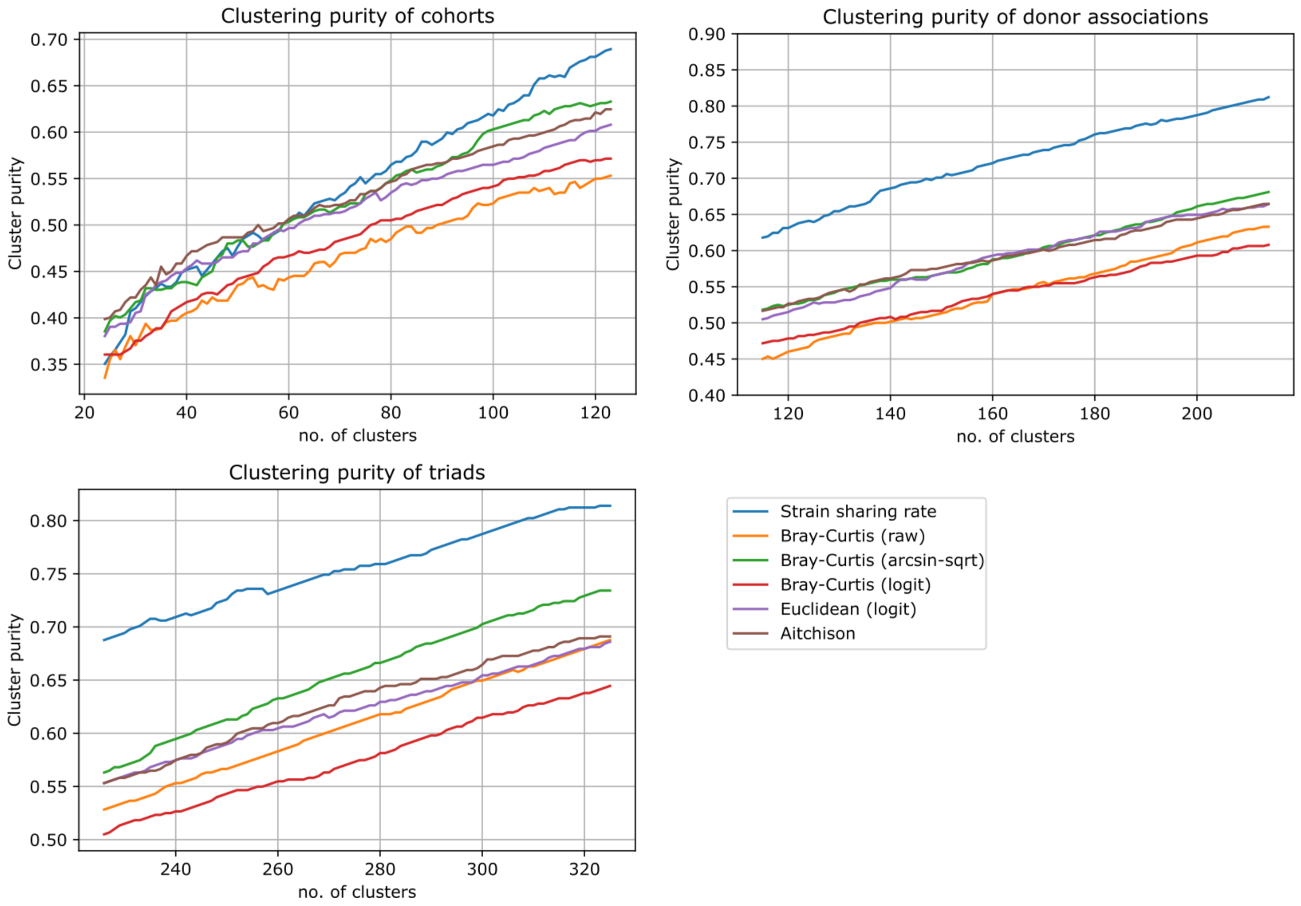

ED Figure 2. **The purity of K-medoids clustering with varying K shows that strain sharing rate outperforms beta diversity measures in clustering by donor associations and by FMT triads.** In clustering by cohorts for the low number of clusters it gets outperformed by Aitchison distance, but catches up as the K increases.

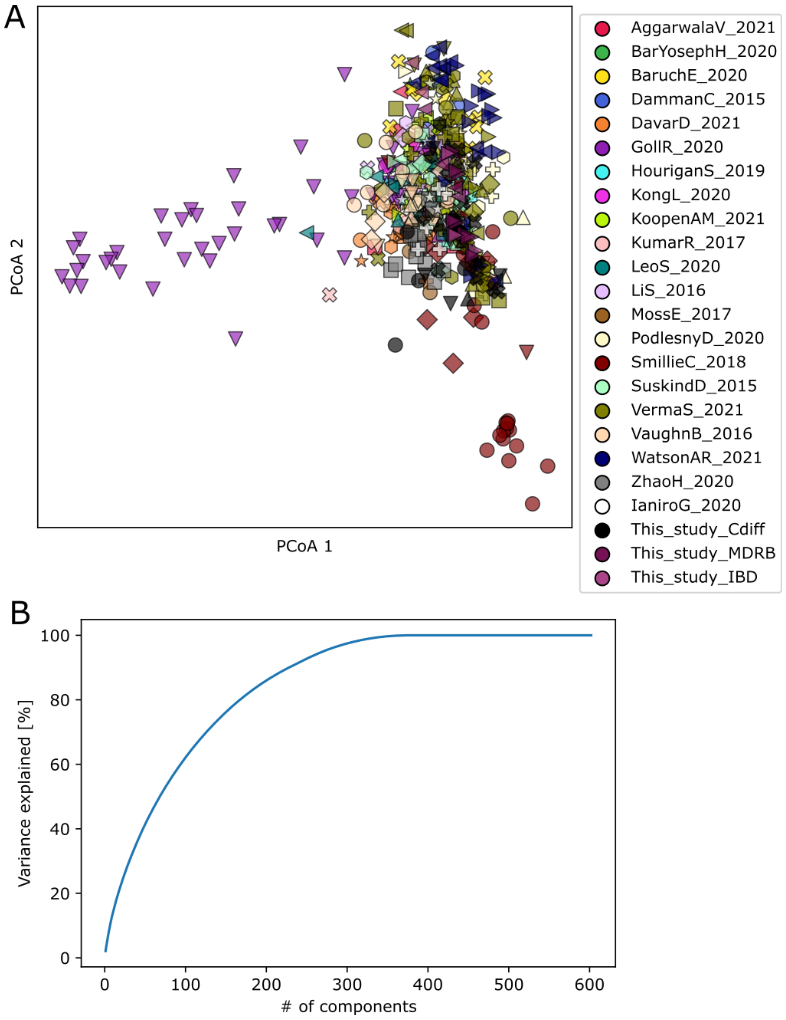

ED Figure 3. PCoA ordination on strain sharing rate distances and variance explained by number of components, suggesting that two dimensions are not sufficient to linearly separate the clusters induced by dataset or donor batch effects. Unique combinations of color and shape correspond to samples associated with one donor subject.

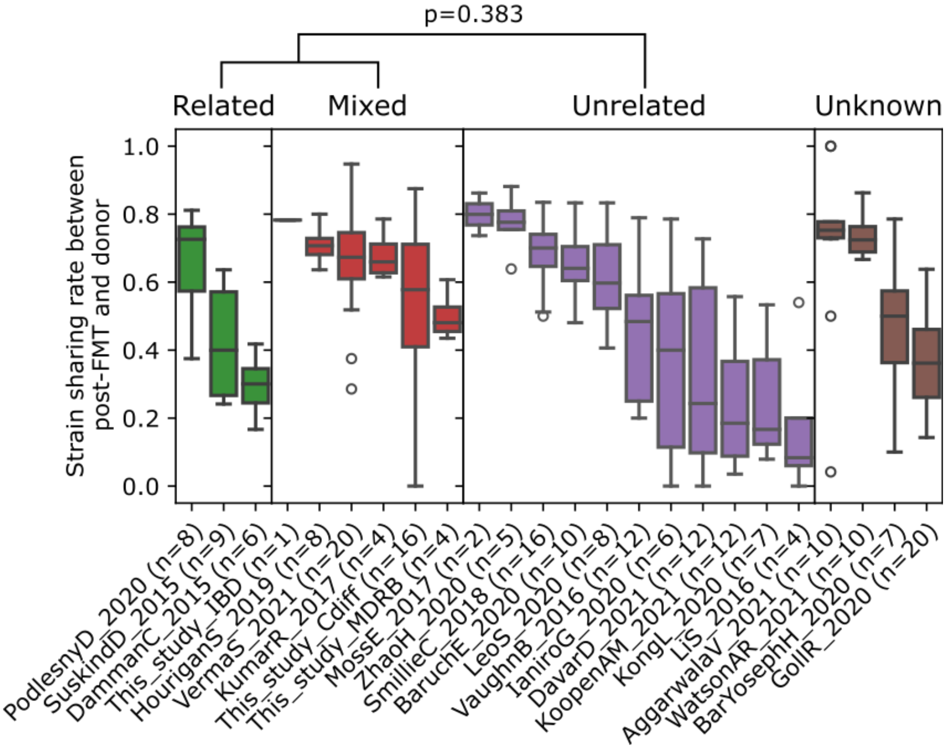

ED Figure 4. Strain sharing rates between donor and post-FMT samples is non-significantly higher in datasets using related or a mixture of related and unrelated donors compared to those using only unrelated donors (related or mixed vs unrelated, permutation test, p=0.383). Box plots are defined as follows: the center line and upper and lower limit of the box correspond to the median, upper quartile and lower quartile respectively. The whiskers are defined by that data point that is at most 1.5 times higher than the upper quartile (upper whisker) or 1.5 times lower than the lower quartile (lower whisker).

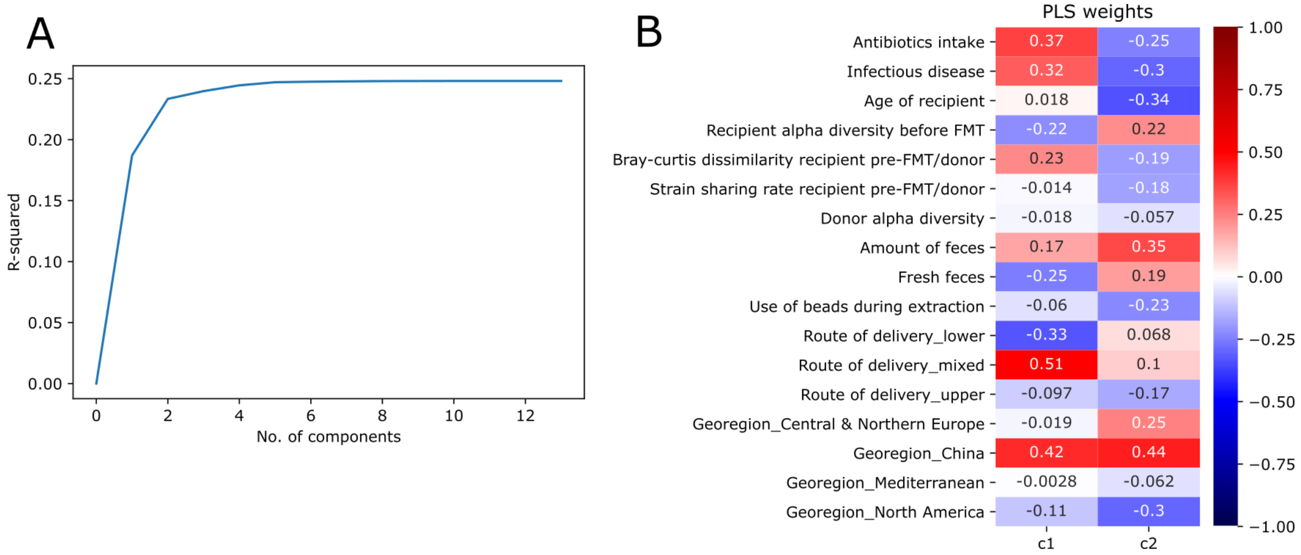

ED Figure 5. Partial least squares regression of various variables of interest against strain engraftment rate. A) Most of the explained variance in strain engraftment rate is covered by the first two components. B) The weights of the variables in the first two components.

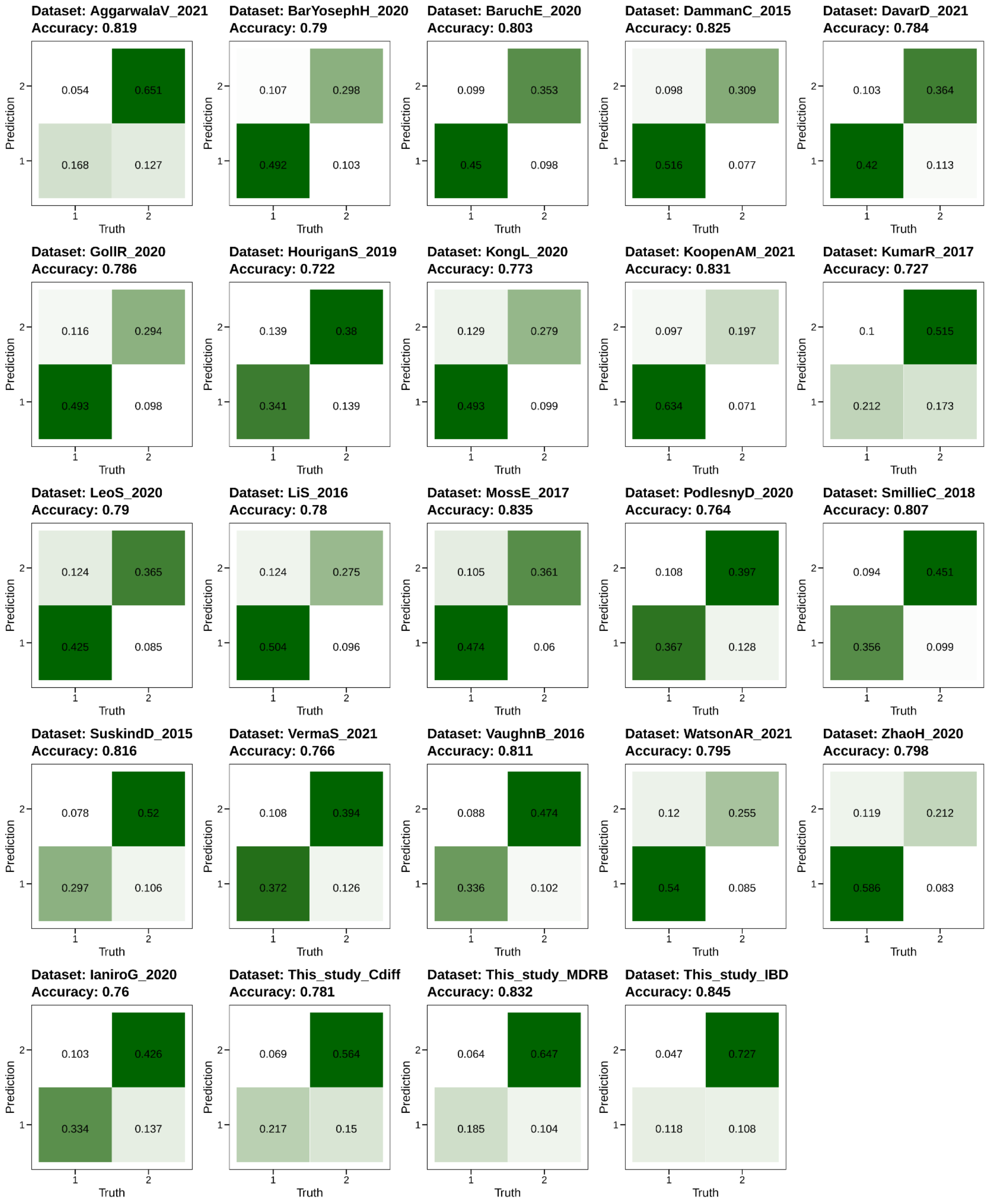

ED Figure 6. Random forest classifier prediction accuracies of post-FMT species presence/absence (CV).

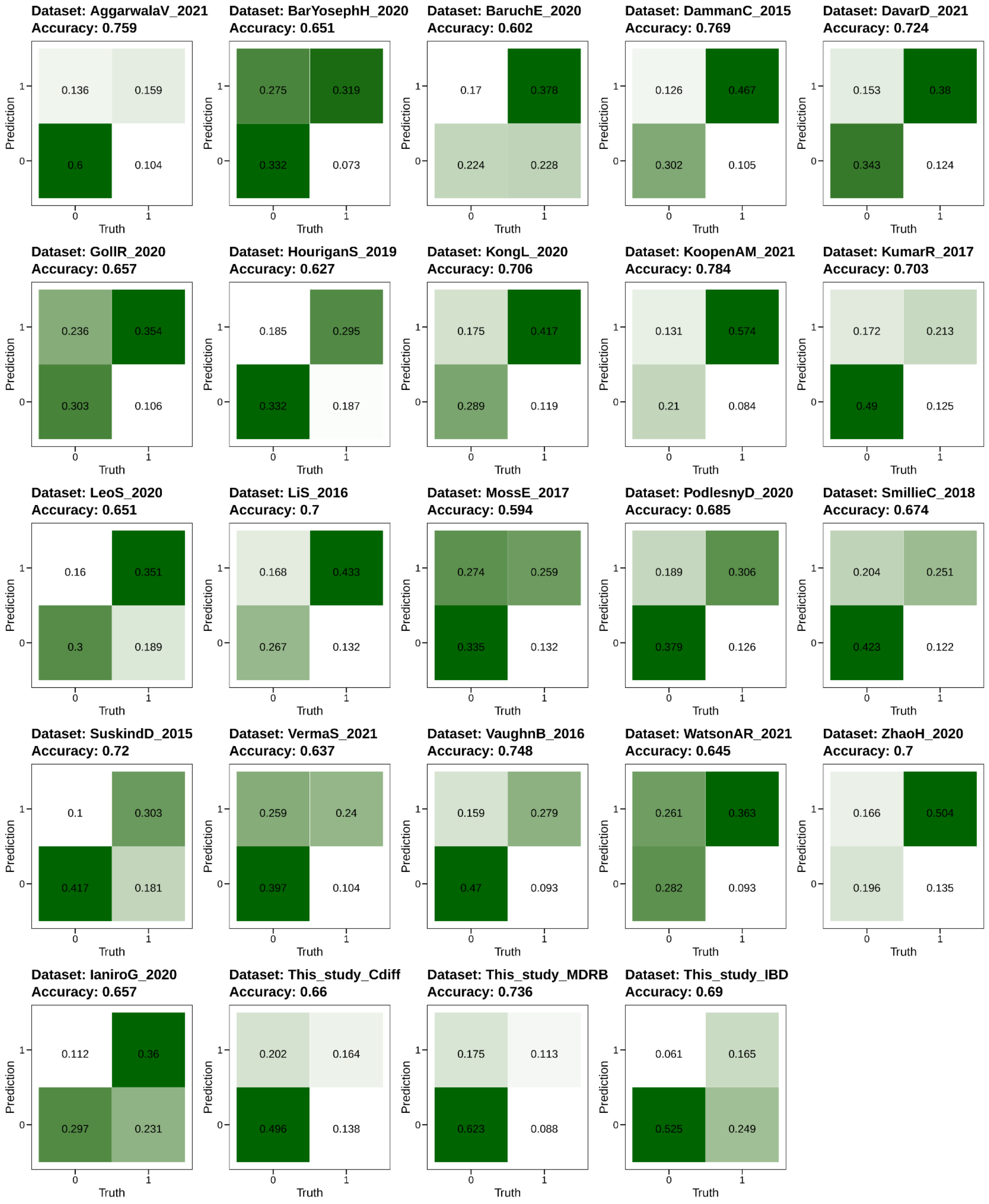

ED Figure 7. Random forest classifier prediction accuracies of post-FMT species presence/absence (LODO).

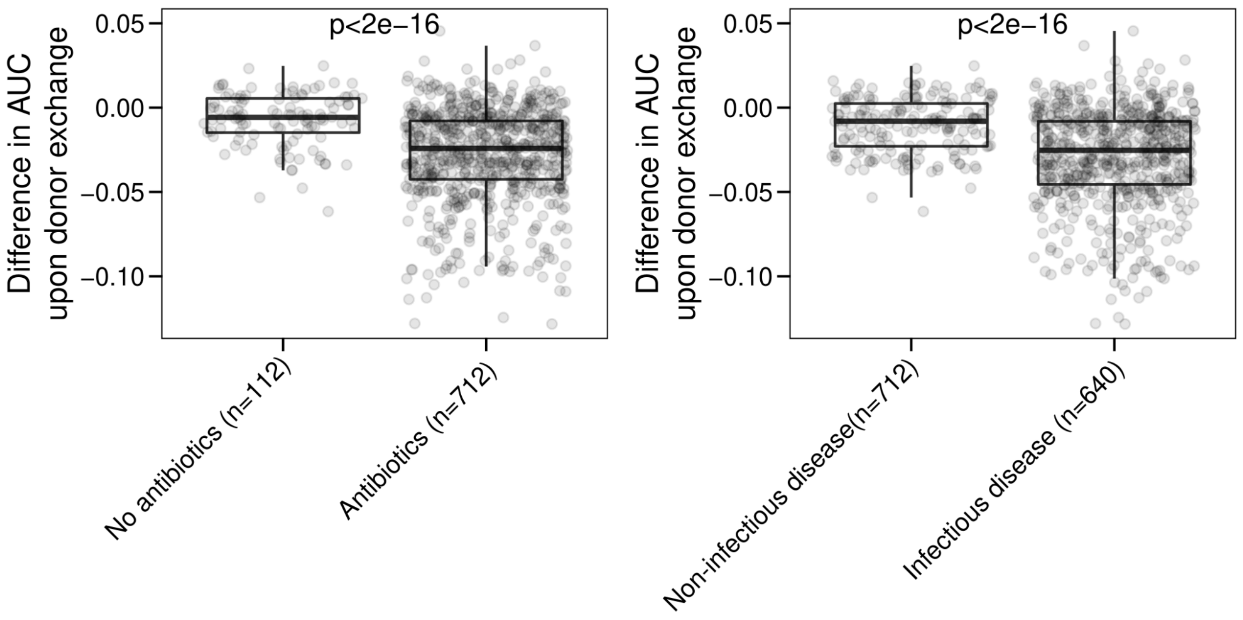

ED Figure 8. Boxplots of the difference in AUC upon simulated donor exchange. Mann-Whitney U-test two-tailed p<2e-16 for both infectious vs. non-infectious disease and antibiotics vs. no antibiotics comparisons. Box plots are defined as follows: the center line and upper and lower limit of the box correspond to the median, upper quartile and lower quartile respectively. The whiskers are defined by that data point that is at most 1.5 times higher than the upper quartile (upper whisker) or 1.5 times lower than the lower quartile (lower whisker).

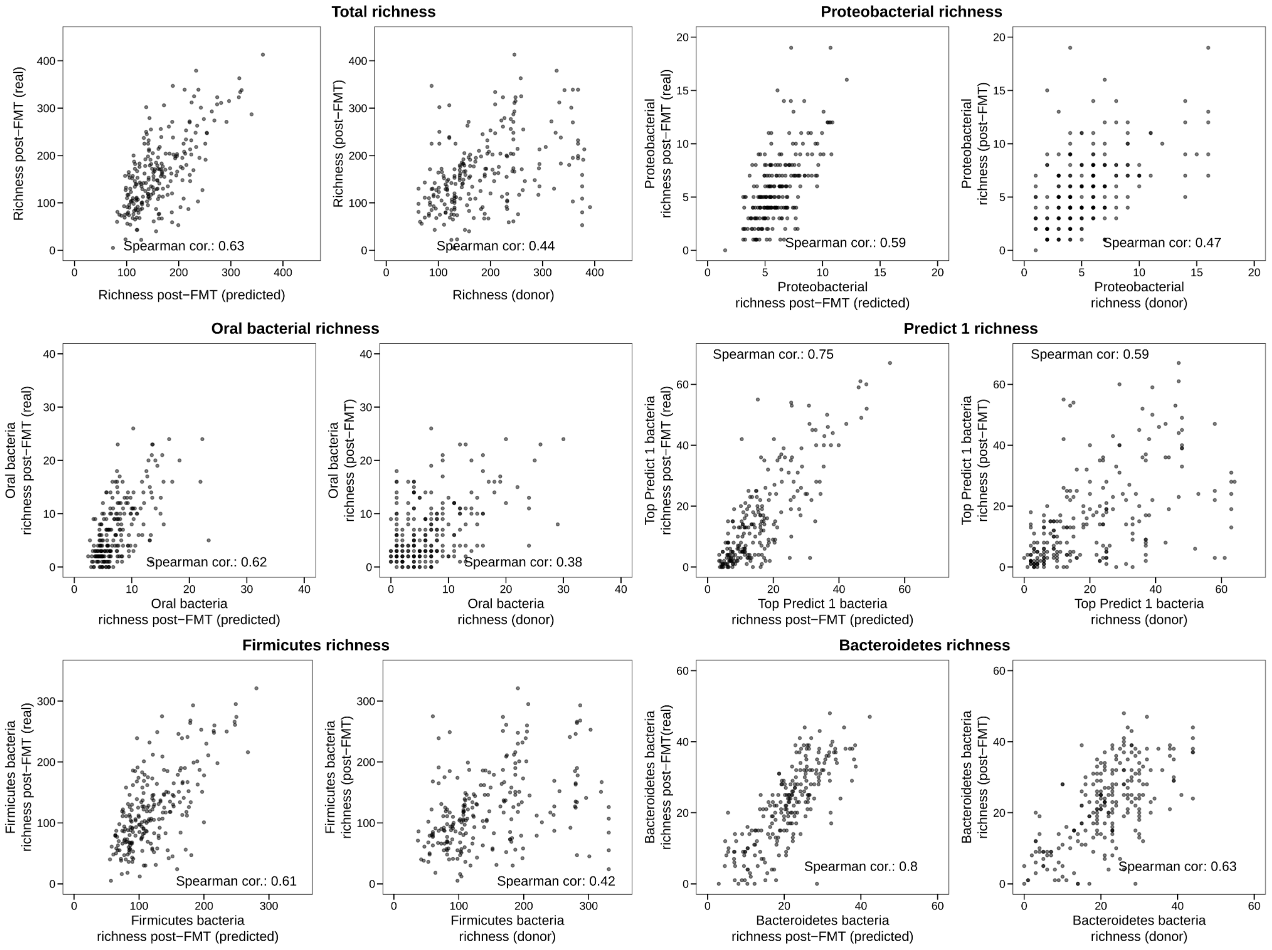

ED Figure 9. Comparisons of the predicted total species richness of bacterial groups in post-FMT samples. Predictions on the y-axis come from the RF classifier, predictions on the x-axis correspond to the cumulative richness in donor samples.

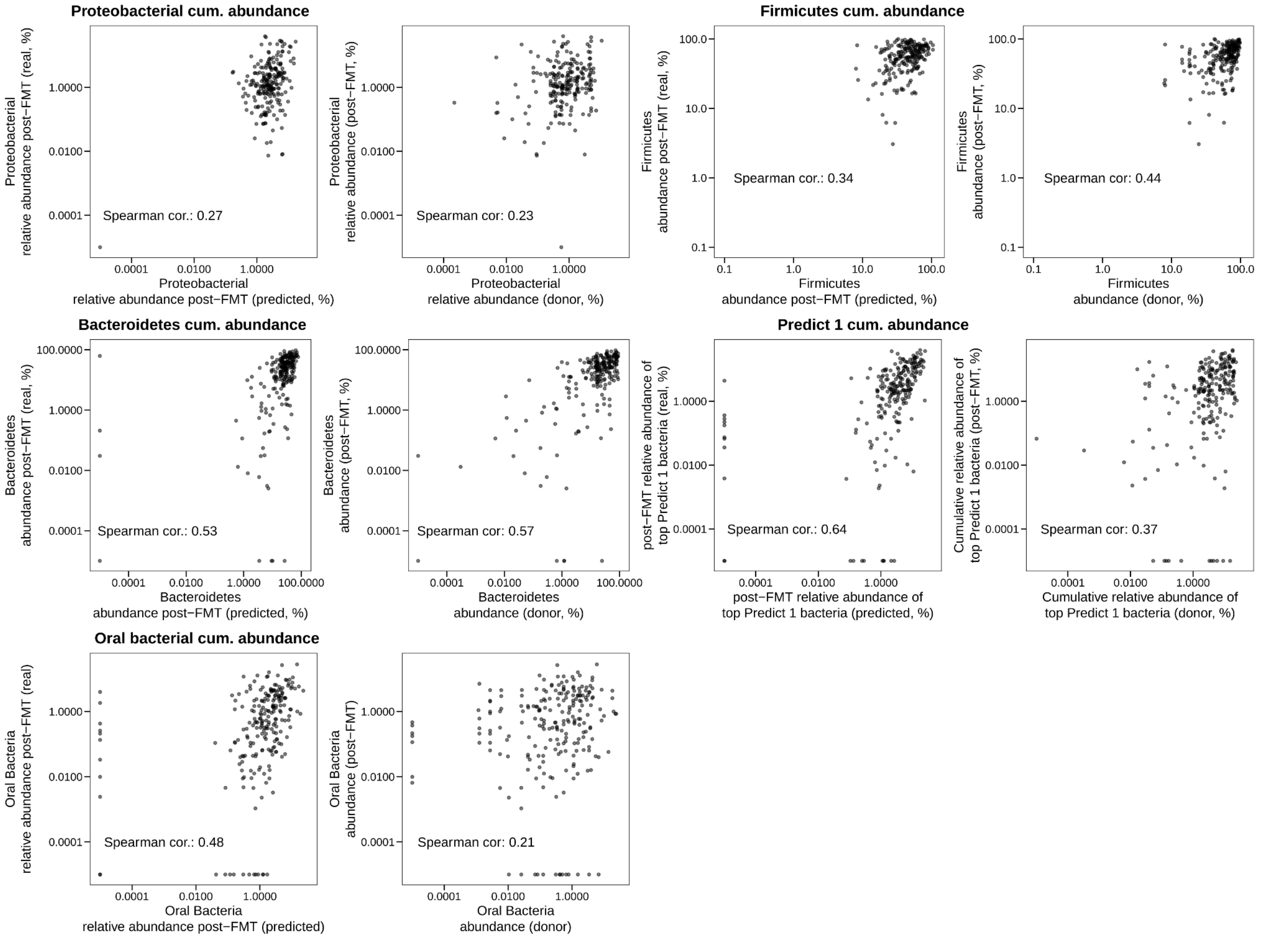

ED Figure 10. Comparisons of the predicted cumulative abundance of bacterial groups in post-FMT samples. Predictions on the y-axis come from the RF regressor, predictions on the x-axis correspond to the cumulative abundance in donor samples.

SUPPLEMENTARY FIGURES AND TABLES

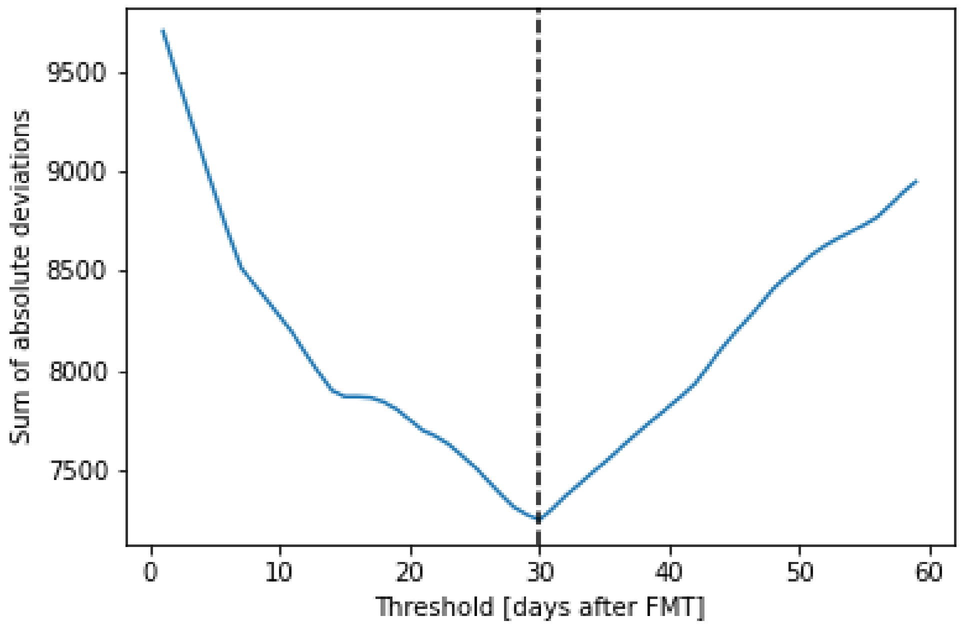

Supplementary Figure 1. Sum of absolute deviations (over all FMT instances) of the sampling time (days) of post-FMT samples. 30 days post-FMT is the value minimizing the sum of absolute deviations.

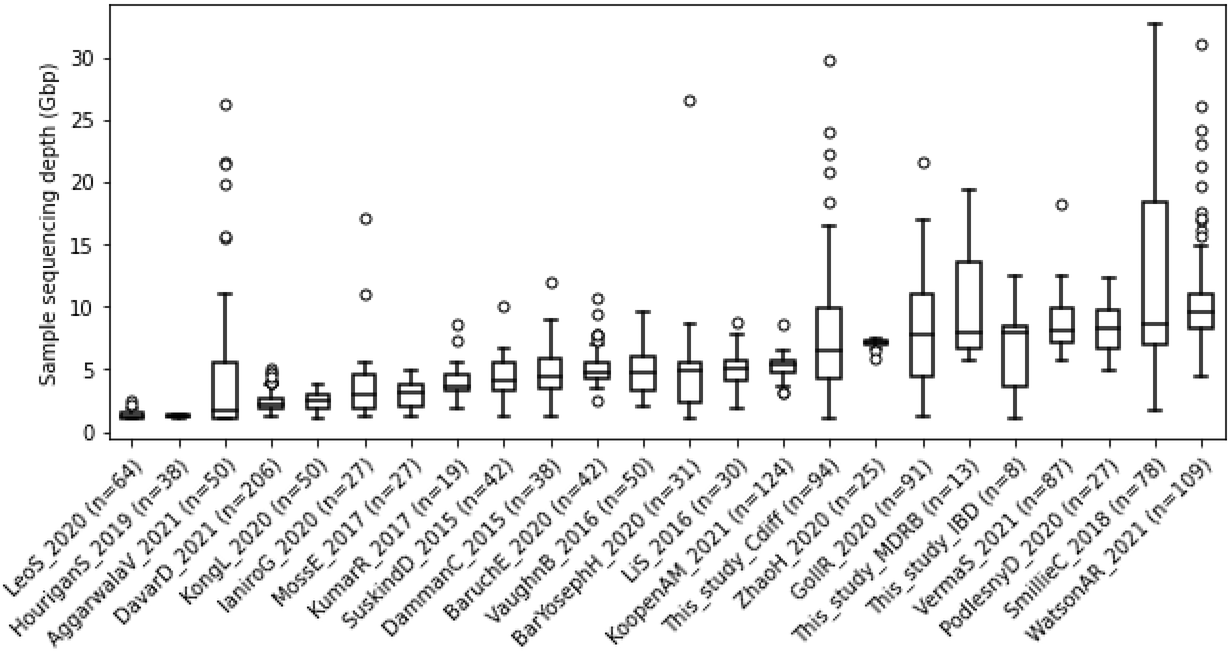

Supplementary Figure 2. Distribution of sequencing depths of all datasets included in this study. Box plots are defined as follows: the center line and upper and lower limit of the box correspond to the median, upper quartile and lower quartile respectively. The whiskers are defined by that data point that is at most 1.5 times higher than the upper quartile (upper whisker) or 1.5 times lower than the lower quartile (lower whisker).

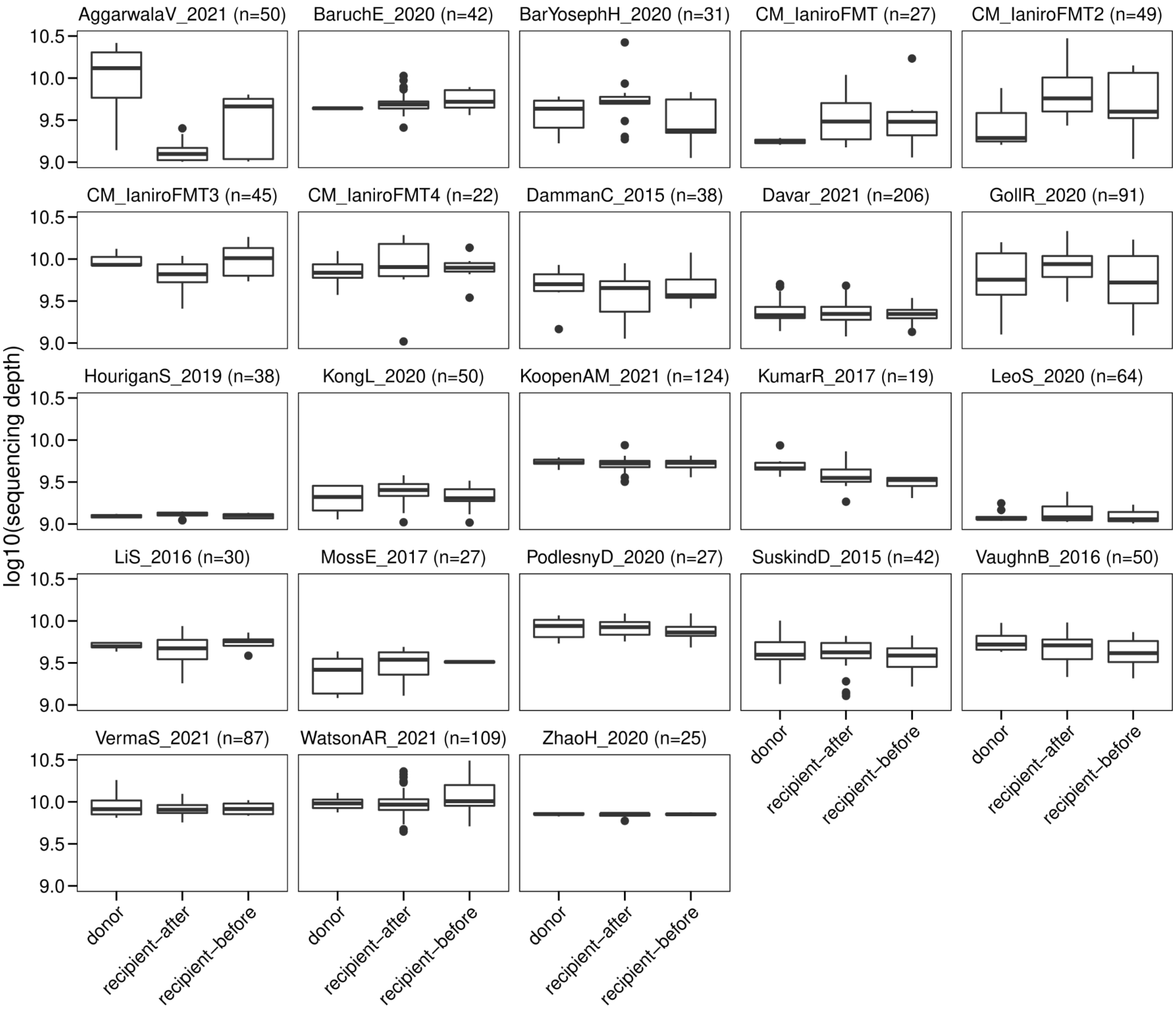

Supplementary Figure 3. Sequencing depths in pre-FMT and post-FMT samples are generally not significantly different. Plotted numbers are unadjusted, two-sided Mann-Whitney U-test p-values. After multiple testing correction (using the BH method) no test remains significant at 10% FDR. Box plots are defined as follows: the center line and upper and lower limit of the box correspond to the median, upper quartile and lower quartile respectively. The whiskers are defined by that data point that is at most 1.5 times higher than the upper quartile (upper whisker) or 1.5 times lower than the lower quartile (lower whisker).

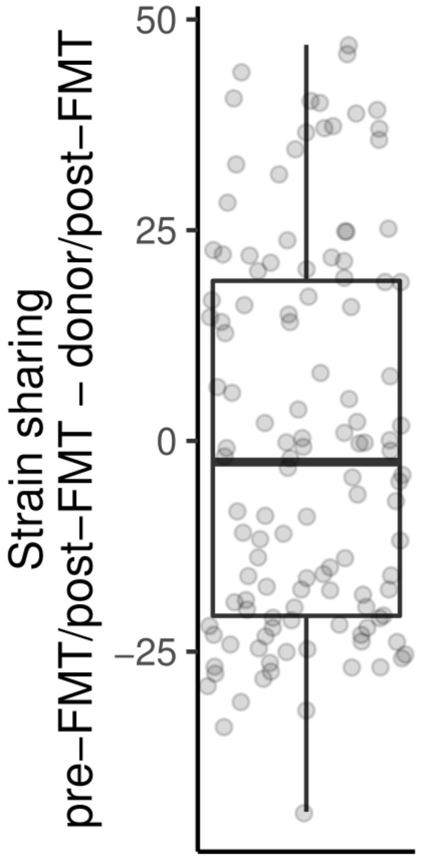

Supplementary Figure 4. Difference in the number of shared strains between pre-FMT/post-FMT and donor/post-FMT sample pairs over all FMT triads, showing the large variation with which recipients retain or take up strains (n=226). A positive number means that the pre-FMT sample shared more strains with the corresponding post-FMT sample than the donor with the corresponding post-FMT sample. Box plots are defined as follows: The center line and upper and lower limit of the box correspond to the median, upper quartile and lower quartile respectively. The whiskers are defined by that data point that is at most 1.5 times higher than the upper quartile (upper whisker) or 1.5 times lower than the lower quartile (lower whisker).

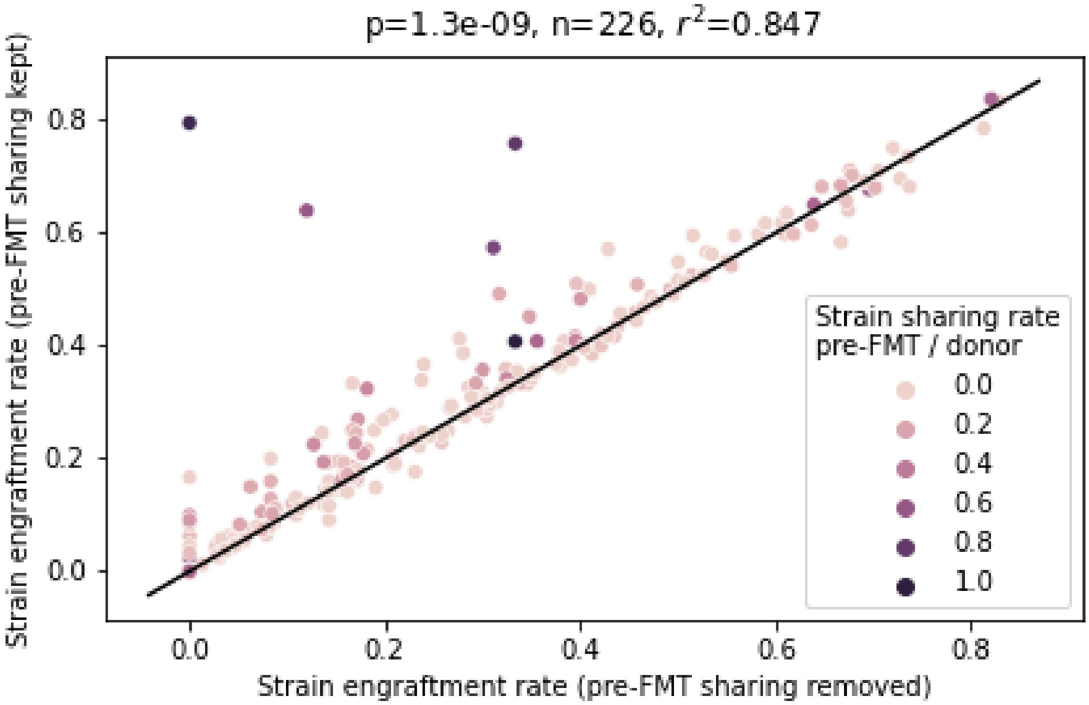

Supplementary Figure 5. Strain engraftment rates would be in many cases overestimated if the amount of strains shared between pre-FMT and donor samples was not subtracted. The difference between the engraftment rates with and without pre-FMT strain sharing removed is significant (Wilcoxon signed-rank test, two-tailed p=1e-9).

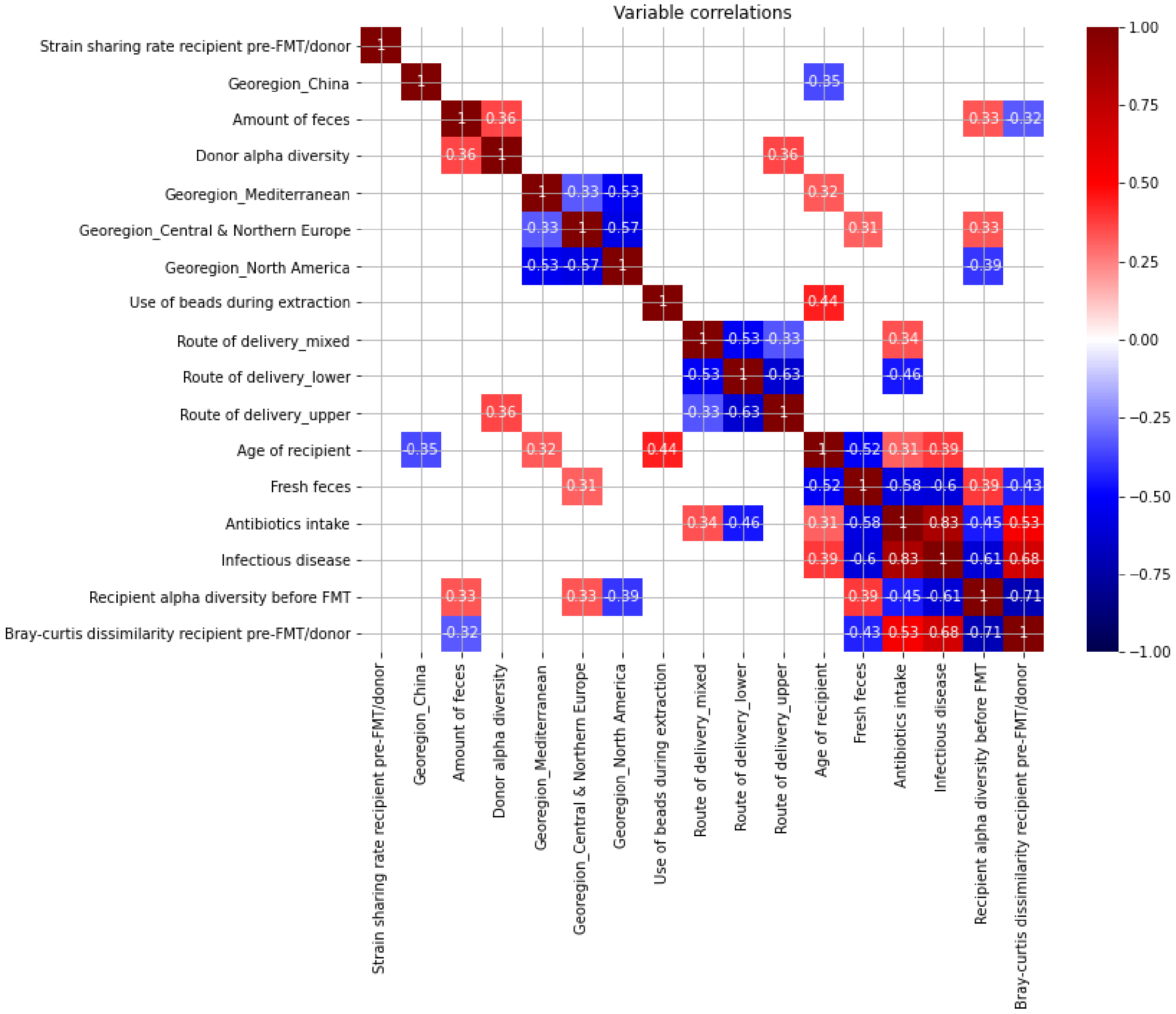

Supplementary Figure 6. Pearson's correlations between variables. Only values of |r|>0.3 are displayed.

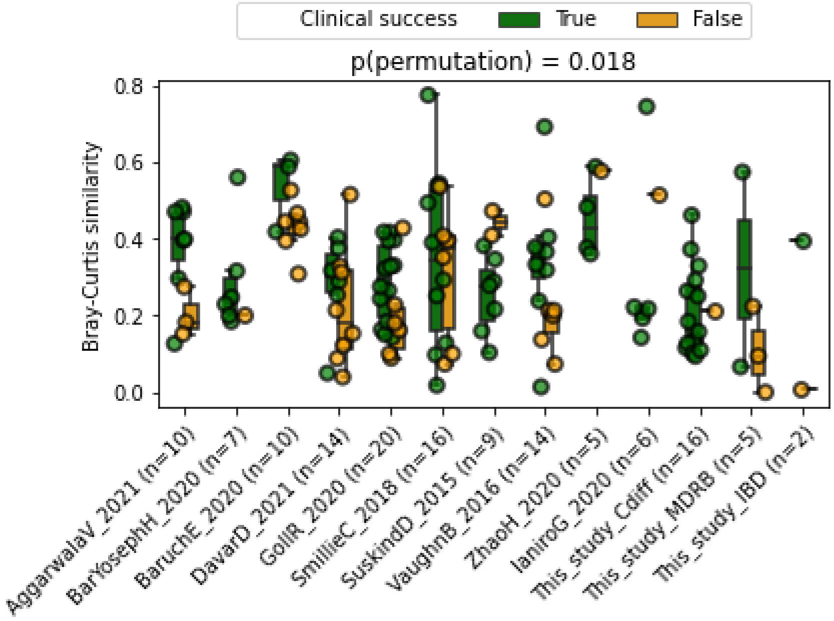

Supplementary Figure 7. Bray-Curtis similarity between cognate post-FMT samples and donor samples by dataset and clinical success. Blocked permutation test two-tailed p=0.018, random effects model p=0.072, Wilcoxon signed-rank test on medians two-tailed p=0.414. Box plots are defined as follows: the center line and upper and lower limit of the box correspond to the median, upper quartile and lower quartile respectively. The whiskers are defined by that data point that is at most 1.5 times higher than the upper quartile (upper whisker) or 1.5 times lower than the lower quartile (lower whisker).

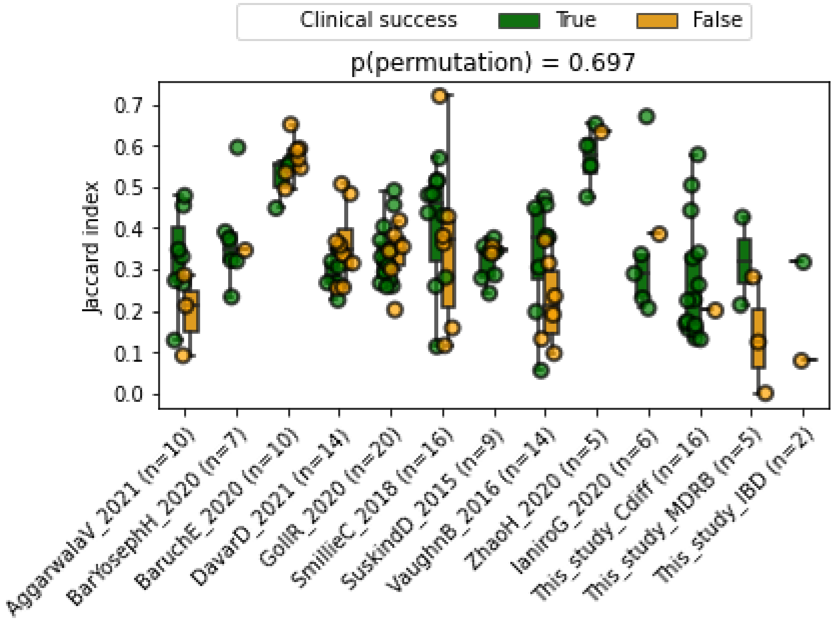

Supplementary Figure 8. Jaccard similarity (based on species presence/absence) between cognate post-FMT samples and donor samples by dataset and clinical success. Blocked permutation test two-tailed p=0.7, random effects model p=0.452, Wilcoxon signed-rank test on medians two-tailed p=0.376. Box plots are defined as follows: the center line and upper and lower

limit of the box correspond to the median, upper quartile and lower quartile respectively. The whiskers are defined by that data point that is at most 1.5 times higher than the upper quartile (upper whisker) or 1.5 times lower than the lower quartile (lower whisker).

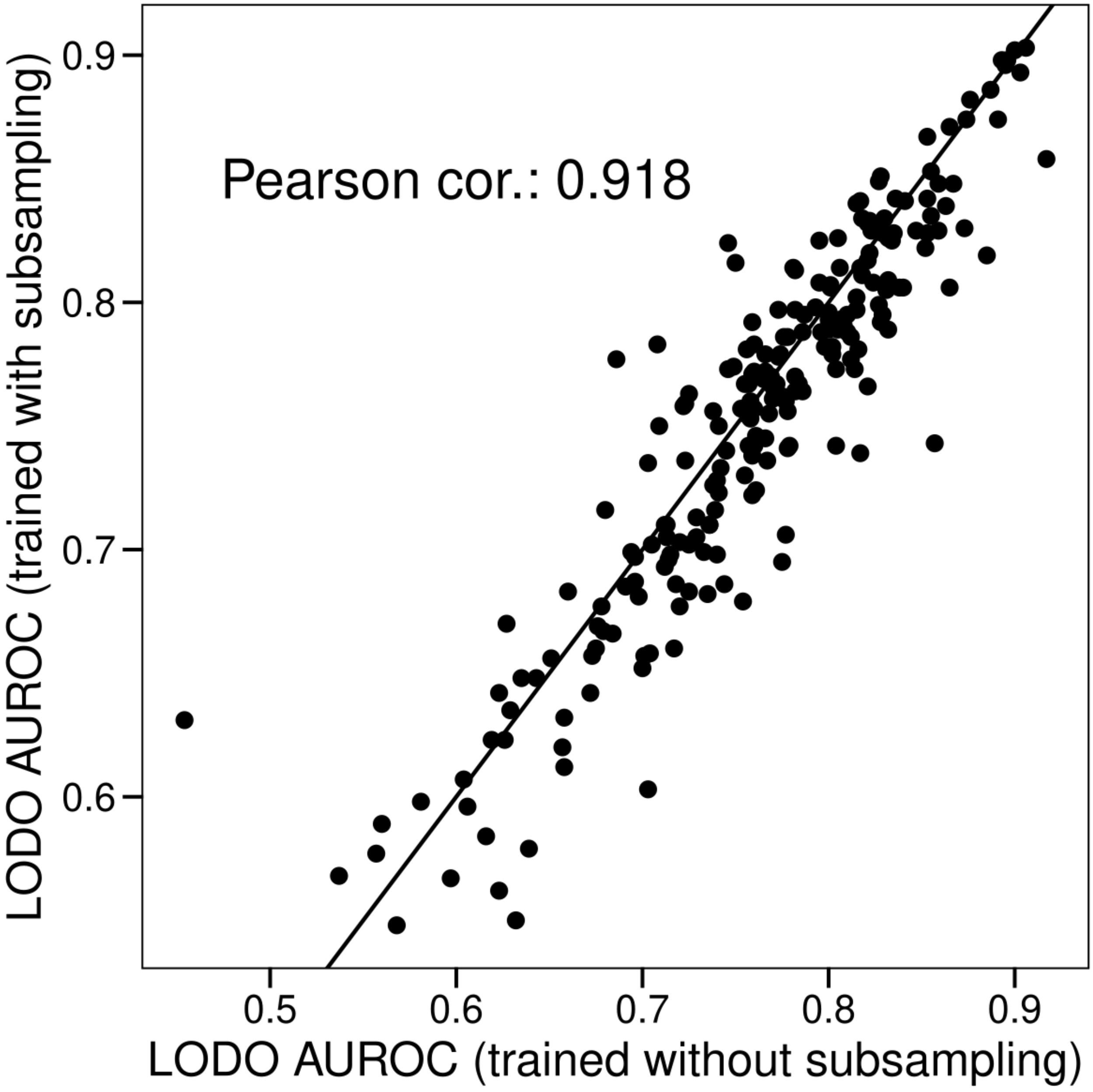

Supplementary Figure 9. LODO AUROC values of models with subsampling each dataset to the number of data points of the smallest dataset and those where we did not use this subsampling strategy.

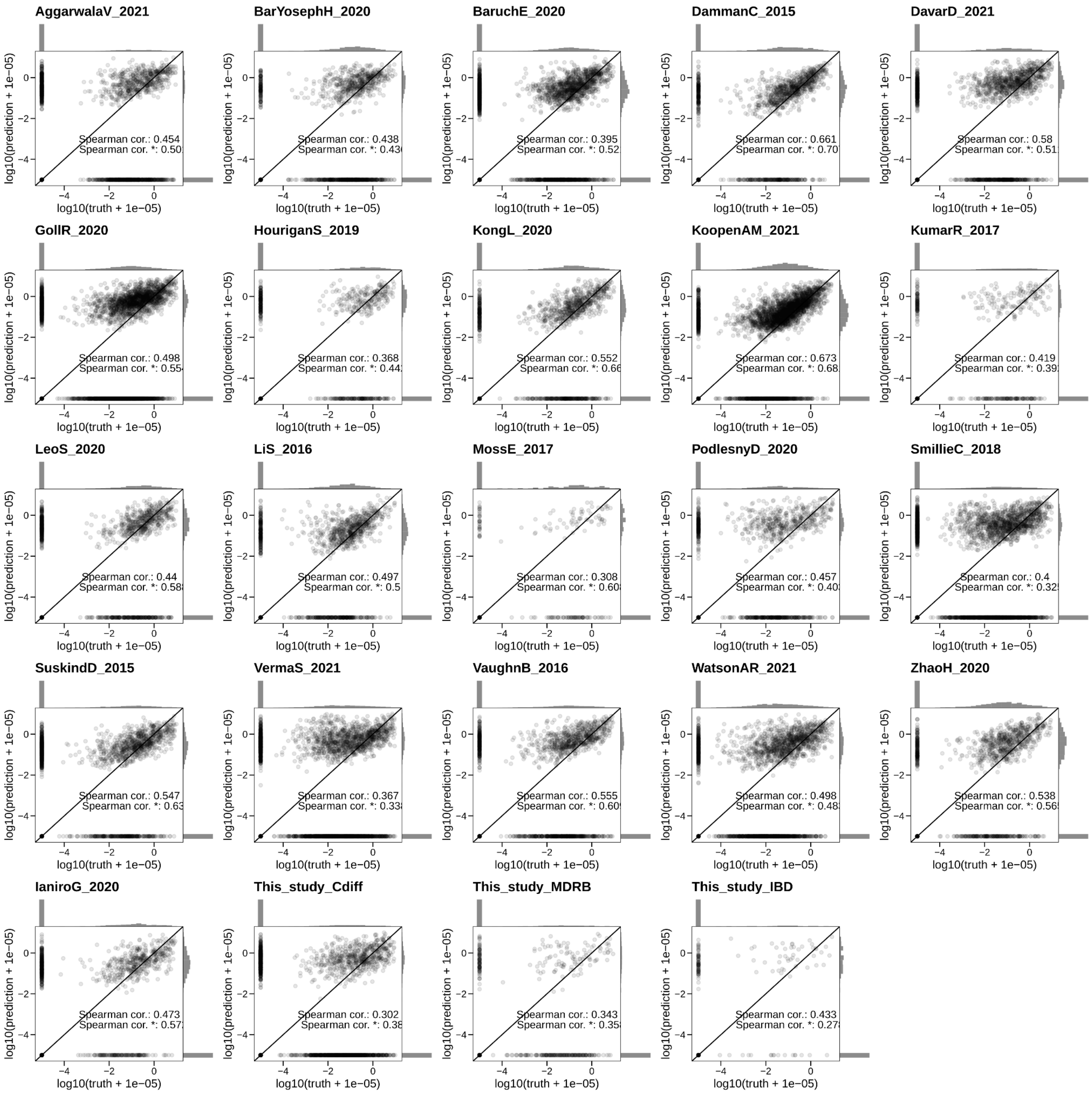

Supplementary Figure 10. Scatterplots by dataset of real vs. predicted bacterial abundances in post-FMT samples. Abundances were predicted using a RF regression model (see Methods). The asterisk designates the Spearman correlation when omitting truly absent species predicted to be absent. Corresponds to Fig. 4G.

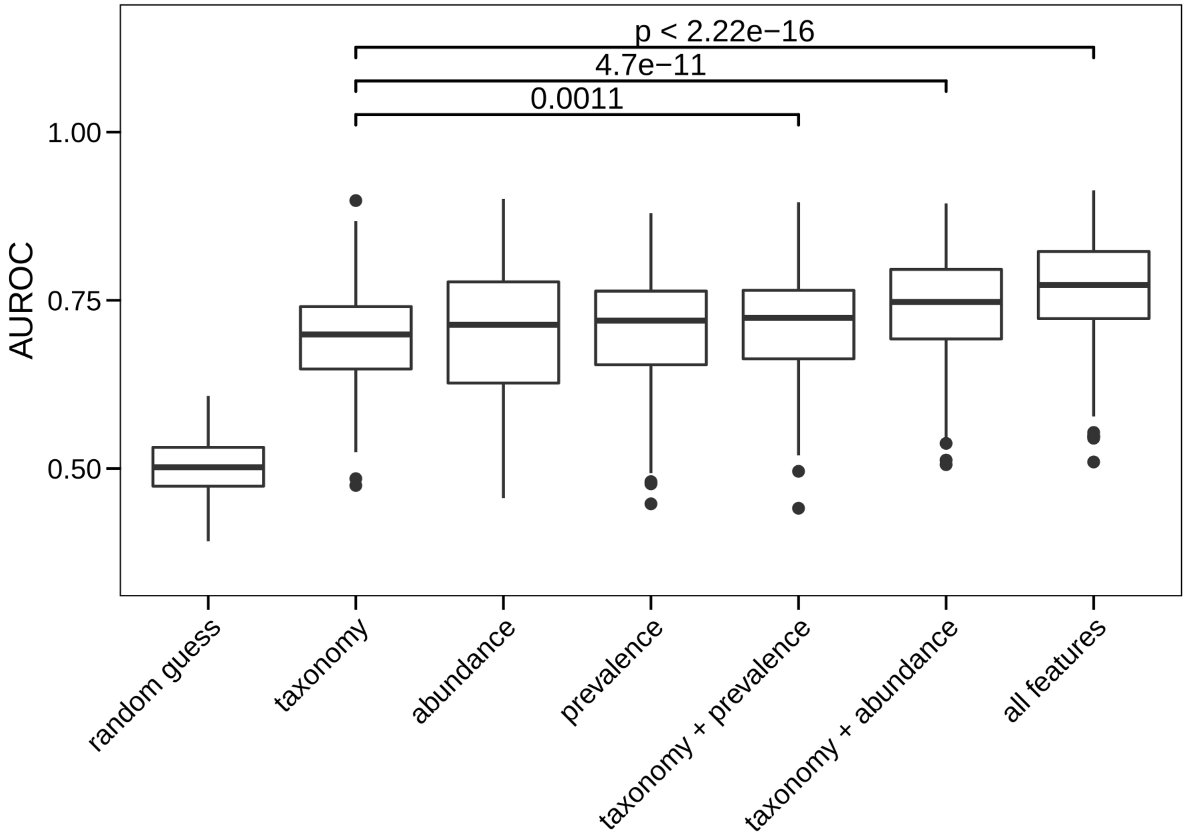

Supplementary Figure 11. LODO AUROC values of post-FMT presence/absence predictions using RF classifiers with varying sets of features (n=24 per group). The plot illustrates that the information content in microbial taxonomic labels is roughly equal to that of abundance or prevalence. Box plots are defined as follows: The center line and upper and lower limit of the box correspond to the median, upper quartile and lower quartile respectively. The whiskers are defined by that data point that is at most 1.5 times higher than the upper quartile (upper whisker) or 1.5 times lower than the lower quartile (lower whisker). P-values were calculated using a two-sided Wilcoxon rank-sum test and were not corrected for multiple comparisons. P-value (taxonomy vs. taxonomy + prevalence) = 0.0011, P-value (taxonomy vs taxonomy + abundance) = 4.7E-11, P-value (taxonomy vs all features) < 2.2E-16.

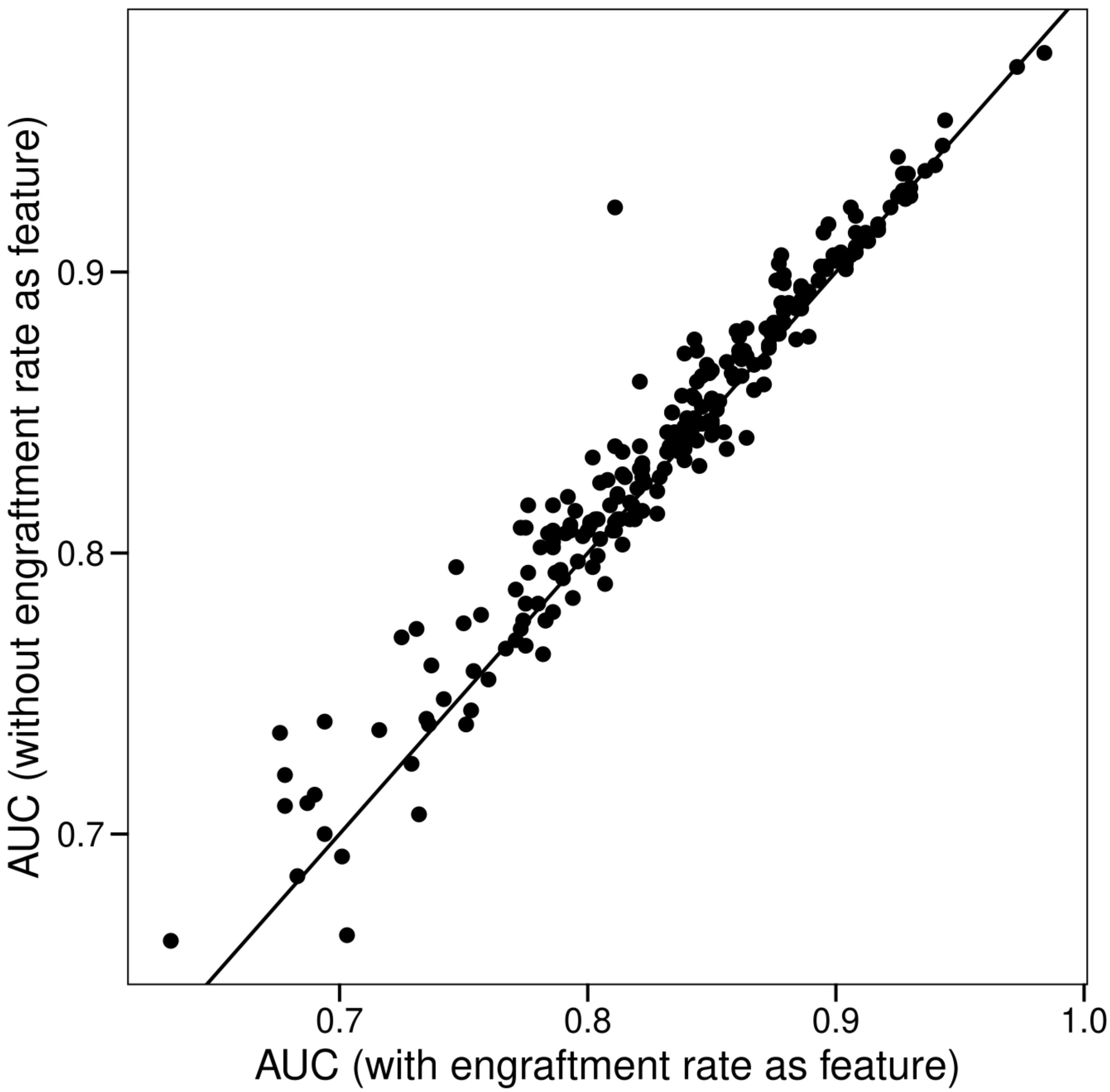

Supplementary Figure 12. CV AUROC values between those models trained containing strain engraftment rate as a feature and those that do not.

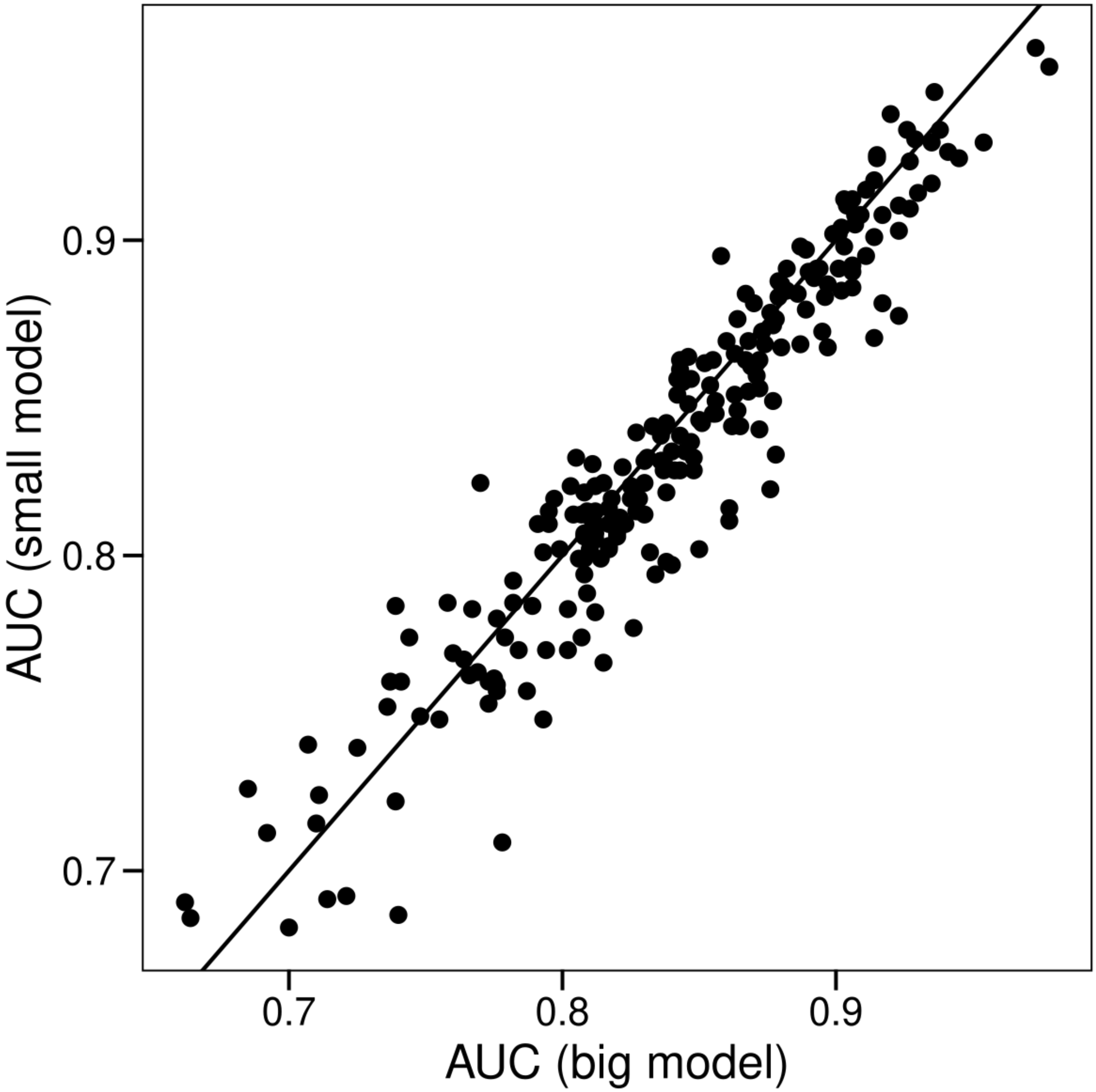

Supplementary Figure 13. CV AUROC values of models reported in Fig. 4 ('small model') and those models where we added also predicted phenotypic traits (Weimann et al. 2016) ('big model').

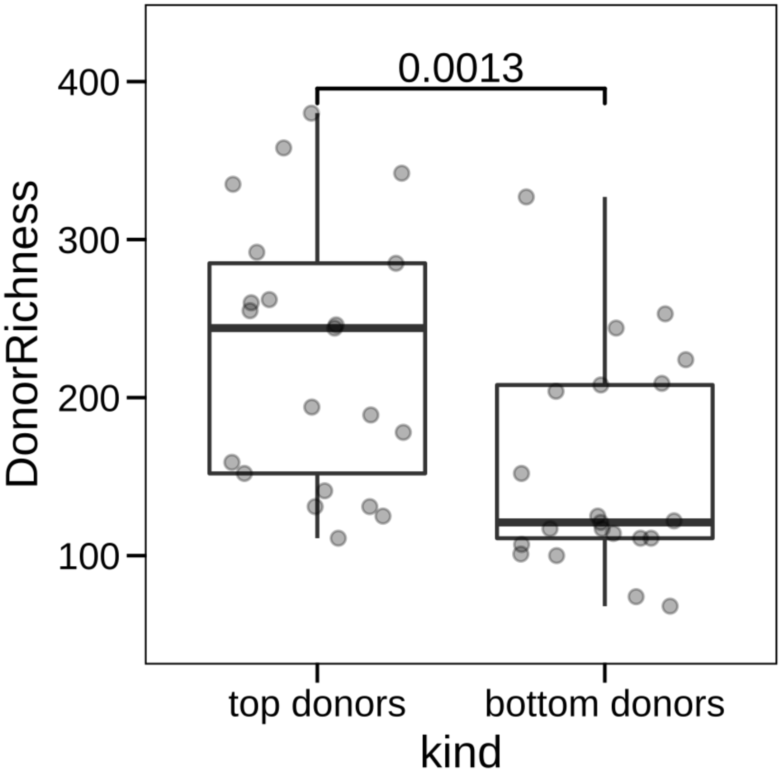

Supplementary Figure 14. Donor species richness of donors that led to a maximum increase in predicted post-FMT richness upon exchange into an FMT triad in each dataset ("top donors") and donors that led to a maximum decrease in predicted post-FMT richness in each dataset ("bottom donors"); n=24 per group. Box plots are defined as follows: the center line and upper and lower limit of the box correspond to the median, upper quartile and lower quartile respectively. The whiskers are defined by that data point that is at most 1.5 times higher than the upper quartile (upper whisker) or 1.5 times lower than the lower quartile (lower whisker). P-values were calculated using a two-sided Wilcoxon rank-sum test.

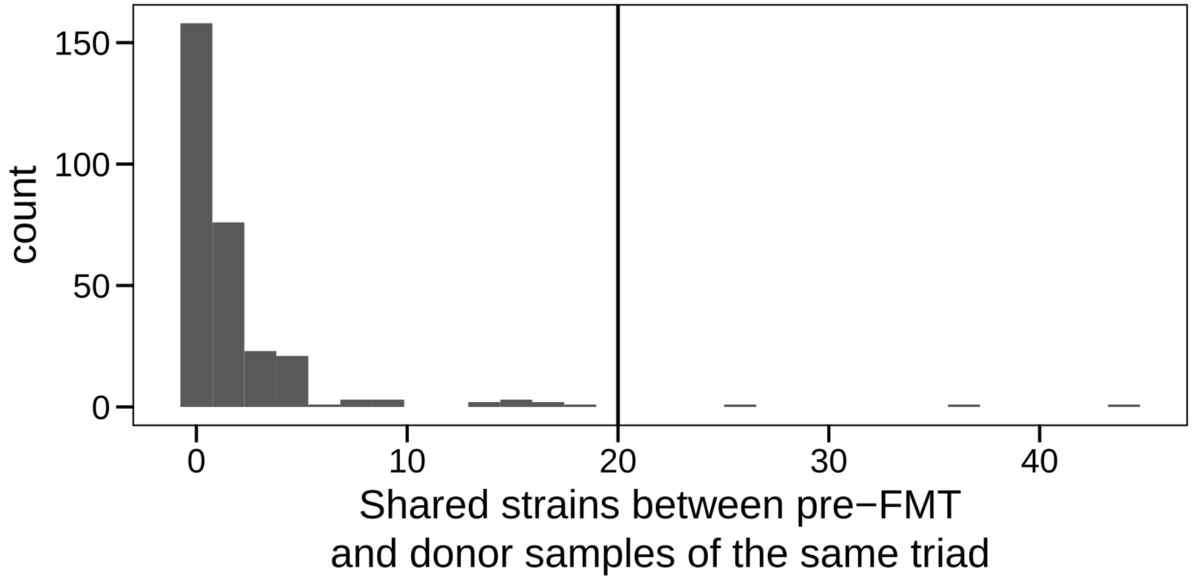

Supplementary Figure 15. Three recipients share an exceptionally high number of strains (>20) with their donor before the FMT, strongly suggesting an error in the metadata.

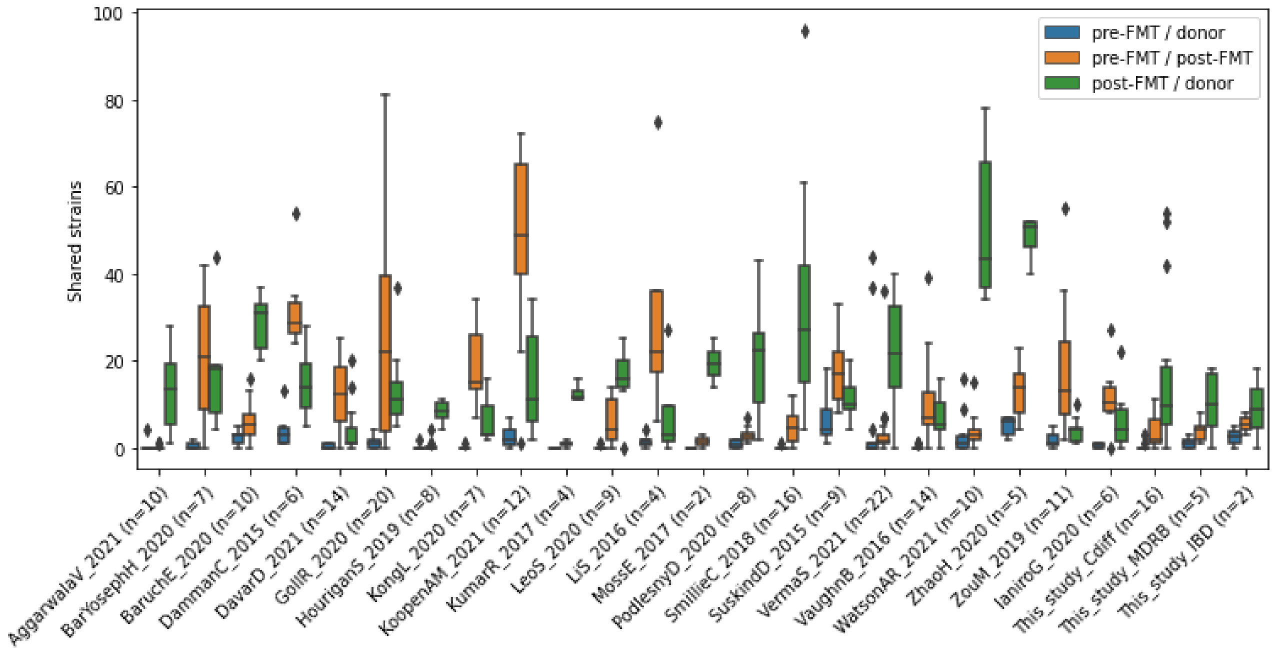

Supplementary Figure 16. Distributions of the number of shared strains within FMT triads by dataset and sample group. Note that The ZouM_2019 dataset is the only dataset in our meta cohort that displays a median of only one shared strain between corresponding post-FMT and donor samples. For this and other reasons, it was excluded from further analyses (see Methods). Box plots are defined as follows: the center line and upper and lower limit of the box correspond to the median, upper quartile and lower quartile respectively. The whiskers are defined by that data point that is at most 1.5 times higher than the upper quartile (upper whisker) or 1.5 times lower than the lower quartile (lower whisker).

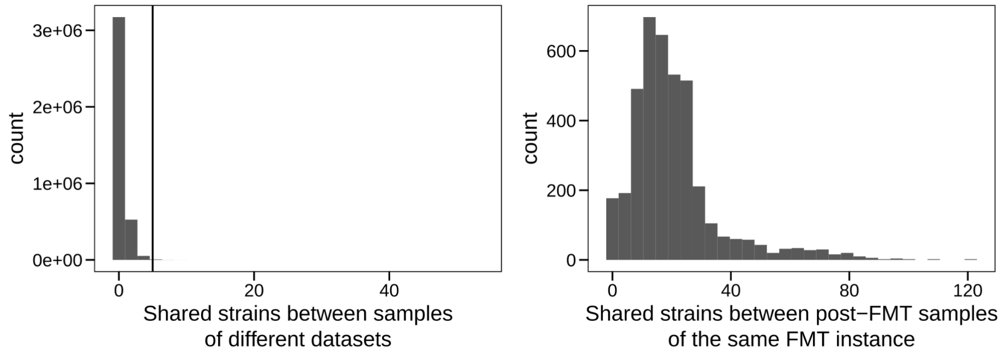

Supplementary Figure 17. Distribution of the number of shared strains between samples coming from different datasets (left) and between post-FMT samples of the same FMT triad (right), showing that unrelated samples rarely share more than 5 strains.

sample name		UWIBD01 D049B0	UWIBD01 D049B1	UWIBD01 D202B0	UWIBD01 D202B1	UWIBD01 D341B0	UWIBD01 D554B0	UWIBD01 D554B1	UWIBD01 D693B0	UWIBD01 D693B1	UWIBD01 D862B0	UWIBD01 D862B1
	subject name	inferred_donor_5	inferred_donor_5	inferred_donor_1	inferred_donor_1	inferred_donor_2	inferred_donor_2	inferred_donor_2	inferred_donor_4	inferred_donor_4	inferred_donor_3	inferred_donor_3
UWIBD01 P016T1	P016	3	0	0	0	41	47	44	1	2	2	3
UWIBD01 P016T2	P016	3	1	0	1	28	27	25	0	2	4	2
UWIBD01 P016T3	P016	0	0	0	0	20	22	20	0	2	1	1
UWIBD01 P081T1	P081	1	1	0	0	0	1	1	39	41	2	1
UWIBD01 P081T2	P081	1	1	0	0	2	1	1	15	14	3	1
UWIBD01 P081T3	P081	1	1	0	0	0	0	0	7	8	2	1
UWIBD01 P224T1	P224	1	0	3	5	0	0	0	1	1	1	0
UWIBD01 P224T2	P224	0	0	5	5	0	0	0	1	1	0	0
UWIBD01 P224T3	P224	1	0	5	5	0	0	0	1	1	1	0
UWIBD01 P271T1	P271	0	0	0	0	2	4	3	2	2	13	8
UWIBD01 P271T2	P271	0	0	0	0	0	0	0	0	0	0	0
UWIBD01 P271T3	P271	0	0	0	0	0	0	0	0	0	0	0
UWIBD01 P279T1	P279	2	1	0	2	30	33	35	4	3	2	0
UWIBD01 P279T2	P279	2	2	0	2	21	29	29	6	6	5	1
UWIBD01 P279T3	P279	1	1	0	1	20	22	19	3	4	3	2
UWIBD01 P788T1	P788	2	2	0	0	0	0	0	2	2	0	0
UWIBD01 P788T2	P788	8	5	0	0	1	1	1	2	2	0	0
UWIBD01 P788T3	P788	5	5	0	0	0	0	0	0	0	0	0

Supplementary Figure 18. Matrix of shared strains between all donor samples and all post-FMT samples in the DammanC_2015 cohort, which was used to infer donor-recipient mapping in this dataset.

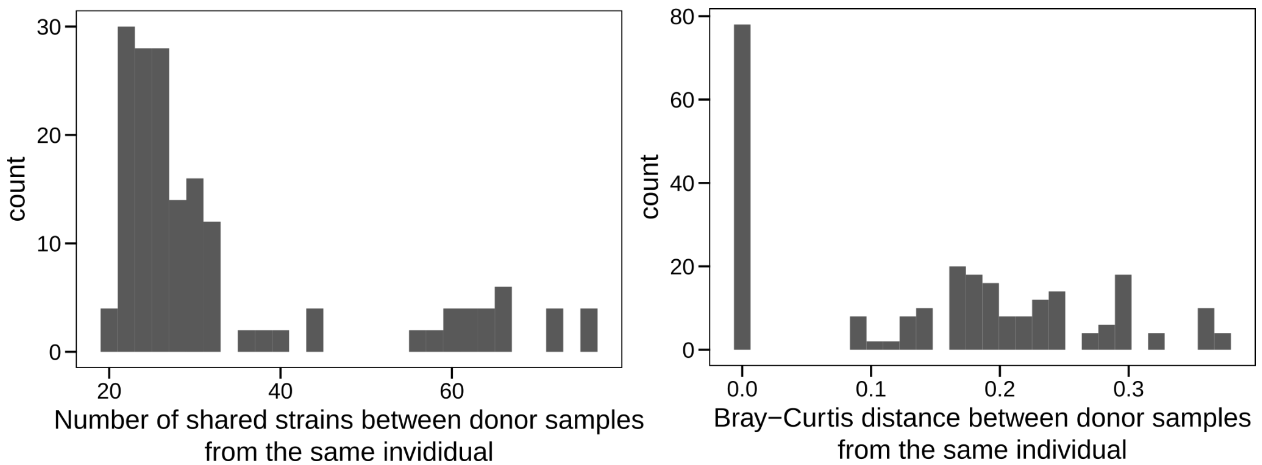

Supplementary Figure 19. Donor samples from the same individual are reasonably stable in terms of species-level composition and strain identity.

Supplementary Table 1. Description and information of all published and novel cohorts used in this meta-analysis.

Supplementary Table 2. Metadata of three novel cohorts used in this meta-analysis.

Supplementary Table 3. Species profiled at strain-level in this analysis and species-wise phylogenetic distances thresholds used to define strain identity.

Supplementary Table 4. Comparison of intra- vs inter-FMT triad distances based on network coordinates. Networks were constructed based on pairwise number of shared strains (see Methods)

Supplementary Table 5. K-medoids clustering with fixed K to the true number of FMT triads, donor subjects and cohorts, respectively. Ad hoc measure is defined as a fraction of post-FMT samples for which the closest sample is the corresponding pre-FMT or donor sample.

Supplementary Table 6. Mann-Whitney U test p-values between all pairs of groups shown in Fig. 1D. p-values were adjusted using the BH method.

Supplementary Table 7. Difference in number of shared strains between cognate pre-FMT/post-FMT and donor/post-FMT sample pairs. Positive number means pre-FMT sample shared more strains with cognate post-FMT sample than the donor.

Supplementary Table 8. Species-wise strain engraftment rates, environment prevalences, and phenotypic trait predictions.

Supplementary Table 9. Presence of Blastocystis subtypes in samples in the meta-cohort, to assess its possible engraftment or retention upon FMT.

Supplementary Table 10. Statistical tests of association of species strain engraftment rates with predicted phenotypic traits and prevalence in different environments.

Supplementary Table 11. Description of the datasets used to assess SGB prevalence in different environments (references and numbers of samples).

Supplementary Table 12. post-FMT presence/absence classifier prediction accuracies in LODO and CV setting.

Supplementary Table 13. Oral prevalence of species found in at least 5% of samples with at least 0.1% abundance in all oral samples of cMD3. Those species with prevalence of at least 20% were considered in the prediction framework.

Supplementary Table 14. Ranked species associations with cardiometabolic health as reported in Supplementary Table 6 of Asnicar et al. 2021. The top 20% (n=80) species were considered in the prediction framework.

Supplementary Table 15. List of MAGs included in the extended SGB database *(external file)*.

Supplementary Table 16. Strain comparison to those found in foods and description of exclusions.
