# Supplementary material for: Variability of strain engraftment and predictability of microbiome composition after fecal microbiota transplantation across different diseases
Source: Nat Med. 2022 Sep 15;28(9):1913–23. doi: 10.1038/s41591-022-01964-3 (PMC9499858; doi:10.1038/s41591-022-01964-3)
Supplement: Supplementary file 2 — Reporting Summary [file 41591_2022_1964_MOESM2_ESM.pdf]

nature portfolio

Corresponding author(s): Gianluca Ianiro, Nicola Segata

Last updated by author(s): Jul 14, 2022

Reporting Summary

Nature Portfolio wishes to improve the reproducibility of the work that we publish. This form provides structure for consistency and transparency in reporting. For further information on Nature Portfolio policies, see our Editorial Policies and the Editorial Policy Checklist.

Statistics

For all statistical analyses, confirm that the following items are present in the figure legend, table legend, main text, or Methods section.

n/a	Confirmed	
☐	☒	The exact sample size (*n*) for each experimental group/condition, given as a discrete number and unit of measurement
☐	☒	A statement on whether measurements were taken from distinct samples or whether the same sample was measured repeatedly
☐	☒	The statistical test(s) used AND whether they are one- or two-sided<br>*Only common tests should be described solely by name; describe more complex techniques in the Methods section.*
☐	☒	A description of all covariates tested
☐	☒	A description of any assumptions or corrections, such as tests of normality and adjustment for multiple comparisons
☐	☒	A full description of the statistical parameters including central tendency (e.g. means) or other basic estimates (e.g. regression coefficient) AND variation (e.g. standard deviation) or associated estimates of uncertainty (e.g. confidence intervals)
☐	☒	For null hypothesis testing, the test statistic (e.g. *F*, *t*, *r*) with confidence intervals, effect sizes, degrees of freedom and *P* value noted<br>*Give P values as exact values whenever suitable.*
☒	☐	For Bayesian analysis, information on the choice of priors and Markov chain Monte Carlo settings
☐	☒	For hierarchical and complex designs, identification of the appropriate level for tests and full reporting of outcomes
☐	☒	Estimates of effect sizes (e.g. Cohen's *d*, Pearson's *r*), indicating how they were calculated

Our web collection on statistics for biologists contains articles on many of the points above.

Software and code

Policy information about availability of computer code

Data collection	No software was used for microbiome data collection.
Data analysis	Newly-generated shotgun metagenomic sequences were pre-processed and quality controlled using the pipeline available at https://github.com/SegataLab/preprocessing and KneadData within bioBakery 3. Species-level profiling was performed using MetaPhlAn 3. Core gene fragments were aligned against the genomes of all SGBs using Bowtie2 (version 2.3.5.1; --sensitive option). Strain profiling was performed with a modified version of StrainPhlAn 3 using the custom SGB marker database. SGB phenotypes were predicted using Traitar (version 1.1.12).<br>Statistical analyses and graphical representations were performed with R (packages curatedMetagenomicData v3.15, igraph 1.2.6, vegan 2.5-7, ggplot2 3.3.5, mlr3 0.10) and Python (packages scikit-learn version 0.24.2, scikit-learn-extra 0.2.0, SciPy 1.8.1).

For manuscripts utilizing custom algorithms or software that are central to the research but not yet described in published literature, software must be made available to editors and reviewers. We strongly encourage code deposition in a community repository (e.g. GitHub). See the Nature Portfolio guidelines for submitting code & software for further information.

March 2021

Data

Policy information about availability of data

All manuscripts must include a data availability statement. This statement should provide the following information, where applicable:

- Accession codes, unique identifiers, or web links for publicly available datasets
- A description of any restrictions on data availability
- For clinical datasets or third party data, please ensure that the statement adheres to our policy

Newly-generated shotgun metagenomics sequencing data are available at the European Nucleotide Archive under accession number PRJEB47909. Metadata are available in Supplementary Table 2 and in curatedMetagenomicData.

Human research participants

Policy information about studies involving human research participants and Sex and Gender in Research.

Reporting on sex and gender	The sex of the study participants is reported in Supplementary Table 1 whenever available. Due to lack of availability in all published studies, this was not added as a covariate in the multivariate models.
Population characteristics	Population characteristics of the 24 studies included in the analysis are reported in Supplementary Tables 1 and 2. 17 cohorts including 147 FMT recipients, 98 donors and 882 samples were included in the analysis, together with three novel cohorts enrolled for this study including 23 recipients, 8 donors and 115 samples.
Recruitment	Three cohorts were newly enrolled, collected and sequenced in the context of this study (Fondazione Policlinico Gemelli IRCCS and Bambino Gesu Children's Hospital, Italy), as detailed in the Methods section. Participants that underwent FMT were recruited in the study, limitting the possibility of self-selection bias.
Ethics oversight	Study procedures of the newly-collected datasets were performed in compliance with the Declaration of Helsinki. Ethical approval was granted by Ethics Committees of Fondazione Policlinico Gemelli IRCCS (ID 3555/2021) and Ospedale Pediatrico Bambino Gesù IRCCS (1107_OPBG_2016). Written informed consent was obtained from all adult participants, and from parents of underaged participants.

Note that full information on the approval of the study protocol must also be provided in the manuscript.

Field-specific reporting

Please select the one below that is the best fit for your research. If you are not sure, read the appropriate sections before making your selection.

☒ Life sciences ☐ Behavioural & social sciences ☐ Ecological, evolutionary & environmental sciences

For a reference copy of the document with all sections, see nature.com/documents/nr-reporting-summary-flat.pdf

Life sciences study design

All studies must disclose on these points even when the disclosure is negative.

Sample size	In this meta-analysis we systematically searched PubMed, Scopus, and ISI Web of Knowledge for potentially eligible studies. In addition, three novel cohorts were newly-sequenced for this study, making it the largest set available. Due to lack of previous data, no power calculation could be performed as part of the study design.
Data exclusions	No data were excluded from the analyses.
Replication	The data included in this study was meta-analysed, no replication was performed.
Randomization	Samples were allocated to FMT triads based on previous published information (metadata of each study) or inferred based on strain sharing patterns when this information was not available. No new groupings were set as part of this analysis.
Blinding	All patients were administered FMT from donors (no placebo). The information on whether samples were from FMT donors or recipients were available to the researchers as this was needed to perform the statistical analysis.

Reporting for specific materials, systems and methods

We require information from authors about some types of materials, experimental systems and methods used in many studies. Here, indicate whether each material, system or method listed is relevant to your study. If you are not sure if a list item applies to your research, read the appropriate section before selecting a response.

Materials & experimental systems

n/a	Involved in the study
☒	☐ Antibodies
☒	☐ Eukaryotic cell lines
☒	☐ Palaeontology and archaeology
☒	☐ Animals and other organisms
☒	☐ Clinical data
☒	☐ Dual use research of concern

Methods

n/a	Involved in the study
☒	☐ ChIP-seq
☒	☐ Flow cytometry
☒	☐ MRI-based neuroimaging
